# Methodological Quality of Prognostic Models for Periodontitis: A Systematic Review and Critical Appraisal

**DOI:** 10.1111/jcpe.70180

**Published:** 2026-08-03

**Authors:** Maryia Karaban, Debora R. Dias, Giuseppe Troiano, Sahar Baniameri, Ishan Ahuja, Divya Joseph, Matteo Serroni, Andrea Ravidà

**Affiliations:** ^1^ Department of Periodontics and Preventive Dentistry School of Dental Medicine, University of Pittsburgh Pittsburgh Pennsylvania USA; ^2^ Department of Biomedical and Neuromotor Sciences University of Bologna Bologna Italy; ^3^ Department of Medicine and Surgery LUM University Casamassima Italy; ^4^ Temple University Maurice H. Kornberg School of Dentistry Philadelphia Pennsylvania USA; ^5^ Unit of Periodontology, Department of Innovative Technologies in Medicine and Dentistry University G. D'Annunzio' of Chieti‐Pescara Chieti Italy

**Keywords:** periodontitis, predictive learning models, prognosis, risk assessment

## Abstract

**Aim:**

To critically appraise the methodological quality and historical evolution of prognostic models for periodontitis.

**Materials and Methods:**

A systematic search was conducted in three databases. Eligible studies were classified as conceptual tools, category‐based tools and data‐driven models. Category‐based and data‐driven models were evaluated with PROBAST. Risk of bias (ROB) was compared across four domains: Participants, Predictors, Outcome and Analysis.

**Results:**

Twenty‐seven studies were included. Prognostic methods evolved from qualitative risk categories and expert‐based classifications towards quantitative, individualised probability estimates generated through multivariable statistical and machine‐learning models, alongside progressive improvements in outcome definition, prediction time and risk expression formats. Conceptual tools were not evaluable under PROBAST due to the absence of empirical data. Category‐based tools showed limitations in predictor handling and validation rather than bias per se. Data‐driven models demonstrated progressive refinement: earlier studies lacked consistent validation, whereas recent models more frequently report internal validation, calibration metrics and external validation. However, all studies except one were classified as high ROB. Domain‐level assessment showed strengths in the Participants domain but persistent limitations in the Analysis domain. Methodological improvements after TRIPOD were modest.

**Conclusion:**

Prognostic models reflect distinct methodological profiles across design type. Conceptual and category‐based frameworks remain clinically relevant historically, while data‐driven models represent the contemporary standard but retain important methodological gaps.

**Trial Registration:**

PROSPERO number: CRD42024525505

## 1 Introduction

Periodontitis is a prevalent chronic inflammatory condition that affects the gingiva, periodontal ligament and alveolar bone (Eke et al. 2012; Kornman et al. 1997). Given its multifactorial aetiology, prognostic tools are pivotal to guide clinical decision making, tailor treatment strategies and improve long‐term outcomes (Armitage 1999). Early systems relied on static clinical parameters such as probing depth (PD) and mobility (Hirschfeld and Wasserman 1978), offering only limited insight into disease dynamics and primarily supporting qualitative risk stratification. Later frameworks incorporated factors like supportive periodontal therapy (Becker et al. 1984), added refined prognostic categories (McGuire and Nunn 1996), emphasised periodontal stability (Kwok and Caton 2007) or targeted complex cases (Miller et al. 2014). More recent strategies have shifted towards multivariable/algorithm‐based frameworks aiming to estimate individualised probabilities of outcomes, such as disease progression or tooth loss. Despite these advancements, models remain heterogeneous in terms of predicted outcomes, prediction time, and output formats, and often lack external validation or adequate methodological rigour (Raittio et al. 2024).

When developing or evaluating prognostic tools, key elements must be clearly defined, including the predicted outcome (and its level and definition), the prediction time and the format of risk estimation (e.g., categories, scores or probabilities) (Raittio et al. 2024). Additionally, TRIPOD (Transparent Reporting of a multivariable prediction model for Individual Prognosis or Diagnosis) (Moons et al. 2015) was developed to improve transparency in prediction model reporting, enhancing reproducibility and facilitating critical appraisal and validation of prognostic models. To further support the systematic evaluation of such models, CHARMS (Checklist for Critical Appraisal and Data Extraction for Systematic Reviews of Prediction Modelling Studies) provides a structured method for critical appraisal (Moons et al. 2014). It helps identify methodological gaps and supports the refinement of models through evaluation of internal and external validation. Complementing CHARMS, PROBAST (Prediction Model Risk of Bias Assessment Tool) specifically assesses the risk of bias (ROB) and applicability of diagnostic and prognostic model studies.

Previous reviews (Chow et al. 2024; Du et al. 2018) have evaluated periodontal prognostic models; however, they did not systematically distinguish between tools based on their methodological approach, including whether they were derived from patient‐level data or based on descriptive frameworks. Nor did they clearly differentiate between risk assessment systems and true prediction models, and did not restrict inclusion to studies reporting original model development. As a result, the foundational steps through which prognostic systems are conceptualised, constructed and justified have not been systematically examined. Therefore, the aim of the present systematic review was to characterise the historical and methodological development of prognostic tools for periodontitis progression and to evaluate their PROBAST evaluability, risk of bias (ROB) in reported predictive performance where applicable and alignment with contemporary methodological standards.

## 2 Materials and Methods

### 2.1 Protocol and Registration

This systematic review was conducted in accordance with the PRISMA guidelines (Moher et al. 2015) and registered in PROSPERO (CRD42024525505).

### 2.2 Research Question

The research question, structured according to the PICOTS framework and consistent with PROBAST guidance, was, ‘In patients diagnosed with periodontitis (Population), which prognostic models or tools (Index) have been developed to predict long‐term tooth loss or disease progression (Timing/Outcome) when applied in clinical practice, and how do these models perform in terms of methodological quality, ROB and adherence to established reporting standards?’.

### 2.3 Eligibility Criteria

Studies were included if they presented original prognostic tools designed for clinical application to estimate the likelihood of periodontitis stability/progression or tooth loss due to periodontitis. Eligible models included scoring systems, nomograms, algorithms or other structured frameworks. Exclusion criteria included narrative or systematic reviews summarising previously published tools, validation or modification of existing models, prediction models (regression analyses) reporting risk factors/indicators for disease progression without developing a prognostic tool or populations without periodontitis.

### 2.4 Search Strategy and Selection Process

See Supporting Information.

### 2.5 Data Collection Process

**FIGURE 1:**
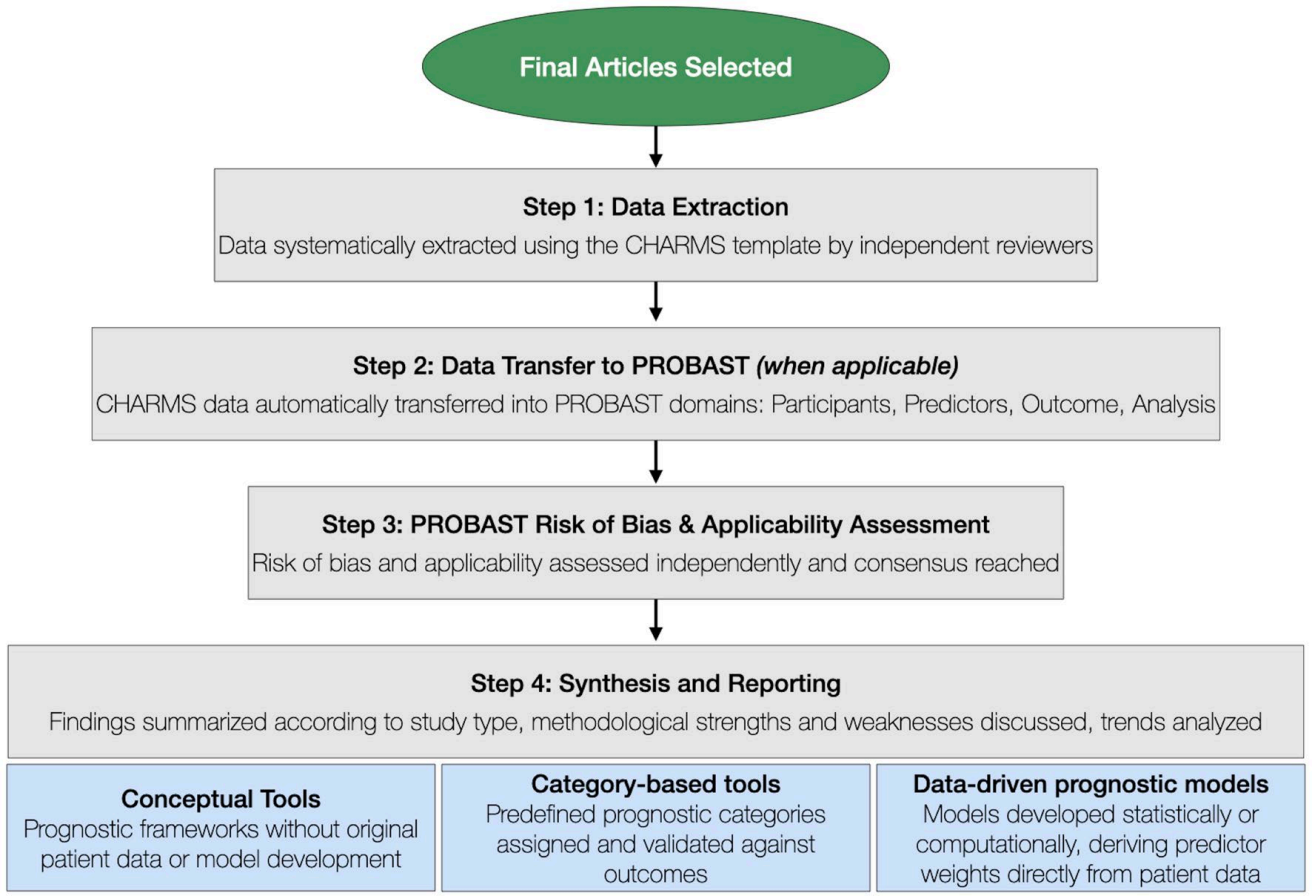
Flowchart illustrating the systematic process used to evaluate studies on periodontal prognosis models and stratification to study groups: (1) Conceptual tools, which proposed prognostic frameworks without original patient data or model development (e.g., expert‐derived risk classifications, theoretical staging systems, or narrative frameworks describing prognostic domains). (2) Category‐based tools, where predefined prognostic categories were assigned and subsequently validated against outcomes (e.g., ‘good/fair/poor’ periodontal prognosis schemes, staging based on mobility/furcation or fixed criteria validated against tooth loss). (3) Data‐driven prognostic models, ranging from conventional statistical approaches without external validation to modern statistical and machine‐learning methods incorporating external validation (e.g., multivariable regression models predicting tooth loss, neural networks trained on clinical datasets or externally validated machine‐learning risk prediction tools).

Data from each selected study were extracted using 11 domains of the CHARMS checklist (Moons et al. 2014). Studies were stratified into three categories: (1) Conceptual tools, which proposed prognostic frameworks without use of original patient data or formal model development; (2) Category‐based tools, in which predefined prognostic categories were assigned and subsequently evaluated against outcomes; and (3) Data‐driven models, developed using patient‐level data through statistical or machine‐learning application (Figure 1). Three reviewers independently classified studies into these categories, with disagreements resolved through discussion. Inter‐rater agreement was high (Fleiss' Kappa [*κ*] = 0.887; *p* < 0.001). All studies were initially evaluated according to key methodological elements for prognostic models: predicted outcome definition and level (patient vs. tooth), prediction time and format of risk estimation (categories, scores, or probabilities).

### 2.6 ROB Assessment

ROB was evaluated using the PROBAST tool (Fernandez‐Felix et al. 2023). Assessment was performed following the integrated CHARMS‐PROBAST approach (Wolff et al. 2019), in which data extracted with CHARMS were directly used to inform PROBAST. Each study was assessed for ROB and applicability across four PROBAST domains ‐ Participants, Predictors, Outcome and Analysis ‐ using predefined responses (Yes [Y], Probably Yes [PY], No Information [NI], Probably No [PN], No [N]; see Supporting Information).

PROBAST was applied according to study design type: (1) Conceptual tools: not eligible for PROBAST assessment due to lack of empirical data/predictive modelling; (2) Category‐based tools: evaluated across PROBAST domains, but Analysis domain findings reflect methodological approach rather than predictive performance bias since no formal multivariable modelling was performed after data collection; (3) Data‐driven models: fully evaluable across all PROBAST domains.

### 2.7 Statistical Analysis

See Supporting Information.

## 3 Results

### 3.1 Study Selection and Characteristics

After screening by title and abstract, there were 1605 records. Thirty‐four articles underwent full‐text review, of which 18 met eligibility criteria. Nine studies were identified through manual search, resulting in 27 studies included in the final analysis (Figure S1). Study characteristics are provided in Supporting Information. Studies were stratified into Conceptual tools (Kwok and Caton 2007; Lang and Tonetti 2003; Grant et al. 1979; Avila et al. 2009; Trombelli et al. 2009; McGowan et al. 2017; Samet and Jotkowitz 2009), Category‐based tools (Hirschfeld and Wasserman 1978; Becker et al. 1984; McGuire 1991; Ghiai and Bissada 1996; Martinez‐Canut and Llobell 2018; Checchi et al. 2002; Page et al. 2002; Fardal et al. 2004; Nibali et al. 2017), and Data‐driven models (Miller et al. 2014; Faggion et al. 2007; Lindskog et al. 2010; Nunn et al. 2012; Ramseier et al. 2019; Ravidà et al. 2020; Shi et al. 2020; Santos et al. 2021; Rahim‐Wöstefeld et al. 2022; Troiano et al. 2023).

### 3.2 Summary of Included Studies

Table 1 summarises the included prognostic tools, stratified by methodological approach. Key elements relevant to interpretation are reported, including the level of analysis, predicted outcome (i.e., what the model aims to predict), prediction time (i.e., when the outcome is expected to occur), model output (i.e., how the prediction is expressed, such as risk categories or probability), time of application and presentation format. Conceptual tools were mainly reported in earlier decades, whereas category‐based and data‐driven models were more frequently reported in later years. Most tools operated at the tooth level and showed marked heterogeneity in outcome definition and prediction time. Quantitative probability‐based outputs were largely restricted to data‐driven models, while earlier approaches predominantly relied on categorical or qualitative risk stratification. Details are in Supporting Information.

**TABLE 1:** Overview of prognostic tools in periodontology, categorised by methodological approach.

Model	Author, year	Level of analysis	Predicted outcome (definition)	Prediction time	Model output (format of risk estimation)	Time of application	Model presentation format	PROBAST
Conceptual tools (prognostic frameworks that did not involve original patient data or model development)	Lang and Tonetti 2003	Patient‐level	Disease progression (NR)	NR	Six parameters → Categories (low, moderate, high)	Re‐eval	Functional diagram (spider web)	Not applicable
	Trombelli et al. 2009	Patient‐level	Disease progression (NR)	NR	Five parameters → Score 1 (low risk) to 5 (high risk)	Re‐eval	Diagram	Not applicable
	Grant et al. 1979	Tooth‐level	Therapeutic (NR)	NR	Qualitative clinical assessment	First appointment	Descriptive (text)	Not applicable
	Kwok and Caton 2007	Tooth‐level	Stability of periodontal supporting tissues (NR)	NR	Qualitative assessment → Categories (favourable, questionable, unfavourable, hopeless)	First appointment	Text/diagram	Not applicable
	Avila et al. 2009	Tooth‐level	Tooth extraction decision	NR	Colour‐coded → Score system (5 categories)	NR	Descriptive (text)	Not applicable
	Samet and Jotkowitz 2009	Tooth‐level	Overall tooth loss	NR	Categories (good, fair, questionable, compromised, nonsalvageable) → Modified by anatomic/iatrogenic factors and patient‐level factors	First appointment	Text/diagram	Not applicable
	McGowan et al. 2017	Tooth‐level	Disease progression (NR)	10 or 5 years	Categories (secure, doubtful, poor, irrational to treat)	First appointment	Table	Not applicable
Category‐based tools (predefined prognostic categories first assigned and subsequently validated against outcome)	Page et al. 2002	Patient‐level	Disease progression (RBL > 2% for all sites)	NR	Multivariable weighted algorithm → Scale (1–5 risk)	First appointment	Computer‐based tool	Applicable
	Hirschfeld and Wasserman 1978	Tooth‐level	NR (perio tooth loss determined by prognosis‐outcome correlation)	NR	Binary (questionable vs. favourable)	First appointment	Descriptive (text)	Applicable
	Becker et al. 1984	Tooth‐level	NR (overall tooth loss determined by prognosis‐outcome correlation)	NR	Categories (good, questionable, hopeless)	First appointment	Descriptive (text)	Applicable
	McGuire 1991	Tooth‐level	NR (overall tooth loss determined by prognosis‐outcome correlation)	NR	Categories (good, fair, poor, questionable, hopeless)	Re‐eval	Descriptive (text)	Applicable
	Ghiai and Bissada 1996	Tooth‐level	NR (overall tooth loss determined by prognosis‐outcome correlation)	NR	Categories (good, fair, guarded, poor, hopeless)	First appointment	Descriptive (text)	Applicable
	Checchi et al. 2002	Tooth‐level	NR (perio tooth loss determined by prognosis‐outcome correlation)	NR	Categories (good, questionable, hopeless)	Re‐eval	Descriptive (text)	Applicable
	Fardal et al. 2004	Tooth‐level	Perio tooth loss	NR	Categories (good, uncertain, poor, hopeless)	Re‐eval	Descriptive (text)	Applicable
	Nibali et al. 2017	Tooth‐level	Overall tooth loss	NR	Categories (good, fair, questionable, unfavourable)	First appointment	Text/diagram	Applicable
	Martinez‐Canut and Llobell 2018	Tooth‐level	Perio tooth survival	NR	Parameters → Scale (0–5 score)	First appointment	Descriptive (text)	Applicable
Data‐driven models (models developed from patient‐level data using statistical or machine‐learning approaches)	Ramseier et al. 2019	Patient‐level	Periodontal stability (PD < 4 mm)	During SPT (dynamic)	Threshold‐based algorithm (residual PPD distribution + modifiers → SPT interval)	Re‐eval	Online tool	Applicable
	Ravidà et al. 2020	Patient‐level	Perio tooth loss (> 1 tooth)	10 years	Nomogram score → Binary (low vs. high risk)	First appointment	Nomogram	Applicable
	Santos et al. 2021	Patient‐level	Perio tooth loss	Early—After diagnosis, pre‐treatment	Equation modelling tooth loss prediction → Probability (0%–100%)	First appointment	Equation	Applicable
	Feher et al. 2025	Patient‐level	Clinical endpoints (≤ 4 sites with PD ≥ 5 mm)	1 year	Probability → Binary (yes or no—threshold 50%)	First appointment	Code available	Applicable
	Faggion et al. 2007	Tooth‐level	Overall tooth survival	Not fixed (~11.8 years)	Categorised probability ranges (9 levels)	First appointment	Graphical model (probability grid)	Applicable
	Lindskog et al. 2010	Tooth‐level	Disease progression (RBL, furcation involvement or angular bony destruction)	Not fixed (~3.8 years)	Score (0–1)	First appointment	Online tool	Applicable
	Nunn et al. 2012	Tooth‐level	Overall tooth survival	Not fixed (10–18 years)	Categories (good, fair, poor, questionable, hopeless)	First appointment	Decision tree (non‐molars and molars)	Applicable
	Rahim‐Wöstefeld et al. 2022	Tooth‐level	Overall tooth loss	10 years	Probability (0%–100%)	First appointment	Model tree	Applicable
	Miller et al. 2014	Molar‐only	Overall molar loss	10, 15, 20, 30 years	Score → Probability (0%–100%)	First appointment	Score‐based index (table)	Applicable
	Shi et al. 2020	Molar‐only	Molar survival	7 years	Nomogram score → Probability (0%–100%)	First appointment	Nomogram	Applicable
	Troiano et al. 2023	Molar‐only	Overall molar loss	10 years	Probability (continuous output; NR)	First appointment	Online tool	Applicable

*Note:* Tools are grouped as conceptual tools, category‐based tools and data‐driven models. For each framework, the table reports the level of analysis (patient‐, tooth‐ or molar‐level), predicted outcome (and its definition as reported), prediction time (i.e., when the outcome is expected to occur), model output (format of risk estimation), time of application and presentation format. Green: clearly reported and methodologically appropriate. Yellow: unclear reporting or methodological limitations. Red: unclear reporting and unclear methodological limitations.

Abbreviation: NR, not reported.

### 3.3 Risk of Bias: PROBAST Domains Assessment

PROBAST was applied to category‐based tools and data‐driven models only.

#### 3.3.1 Domain 1: Participants

##### 3.3.1.1 Were Appropriate Data Sources Used (e.g., Cohort, RCT or Nested Case–Control Study Data)?

**FIGURE 2:**
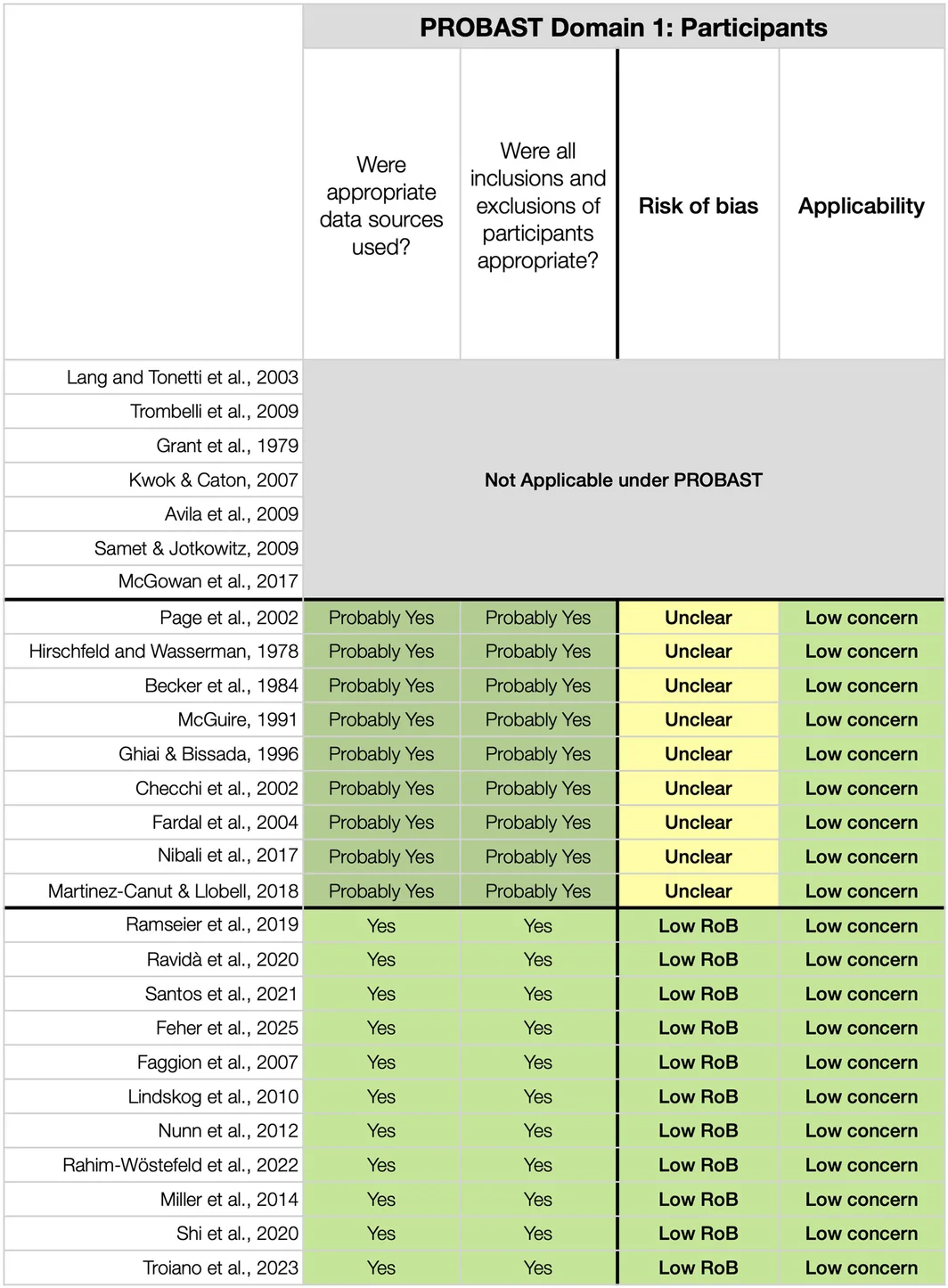
ROB assessment in the Participants domain. Of 27 included studies, 11 were judged as ‘low’ risk, 9 as ‘unclear’ and 7 as ‘not applicable’. Applicability concerns were ‘low concern’ for 20 studies and ‘not applicable’ for the 7 conceptual tools, which did not enrol empirical participants. A black horizontal line separates the three study categories: conceptual, category‐based, and data‐driven. Colour coding: green = low risk/concern, yellow = unclear risk/concern, red = high risk/concern, grey = not applicable.

Of 20 studies reviewed, 11 (Miller et al. 2014; Faggion et al. 2007; Lindskog et al. 2010; Nunn et al. 2012; Ramseier et al. 2019; Ravidà et al. 2020; Shi et al. 2020; Santos et al. 2021; Rahim‐Wöstefeld et al. 2022; Troiano et al. 2023; Feher et al. 2025) used appropriate data sources for predictor assessment (all data‐driven models; Figure 2). Most were retrospective cohorts, while two (Lindskog et al. 2010; Rahim‐Wöstefeld et al. 2022) were prospective and one (Troiano et al. 2023) included both prospective and retrospective external validation cohorts. All category‐based tools were judged PY (probably yes) because they used clinical cohorts to evaluate predefined prognostic classifications rather than to develop formal prediction models.

##### 3.3.1.2 Were All the Inclusions and Exclusions of Participants Appropriate?

Appropriate inclusion and exclusion criteria were reported in all data‐driven models (Figure 2). Category‐based studies were judged PY, as these patient datasets were not used to develop prediction models. Most of these studies required patients to be enrolled in long‐term supportive periodontal therapy (SPT). Exclusion criteria typically involved incomplete records.

#### 3.3.2 Domain 2: Predictors

##### 3.3.2.1 Were Predictors Defined and Assessed in a Similar Way for All Participants?

**FIGURE 3:**
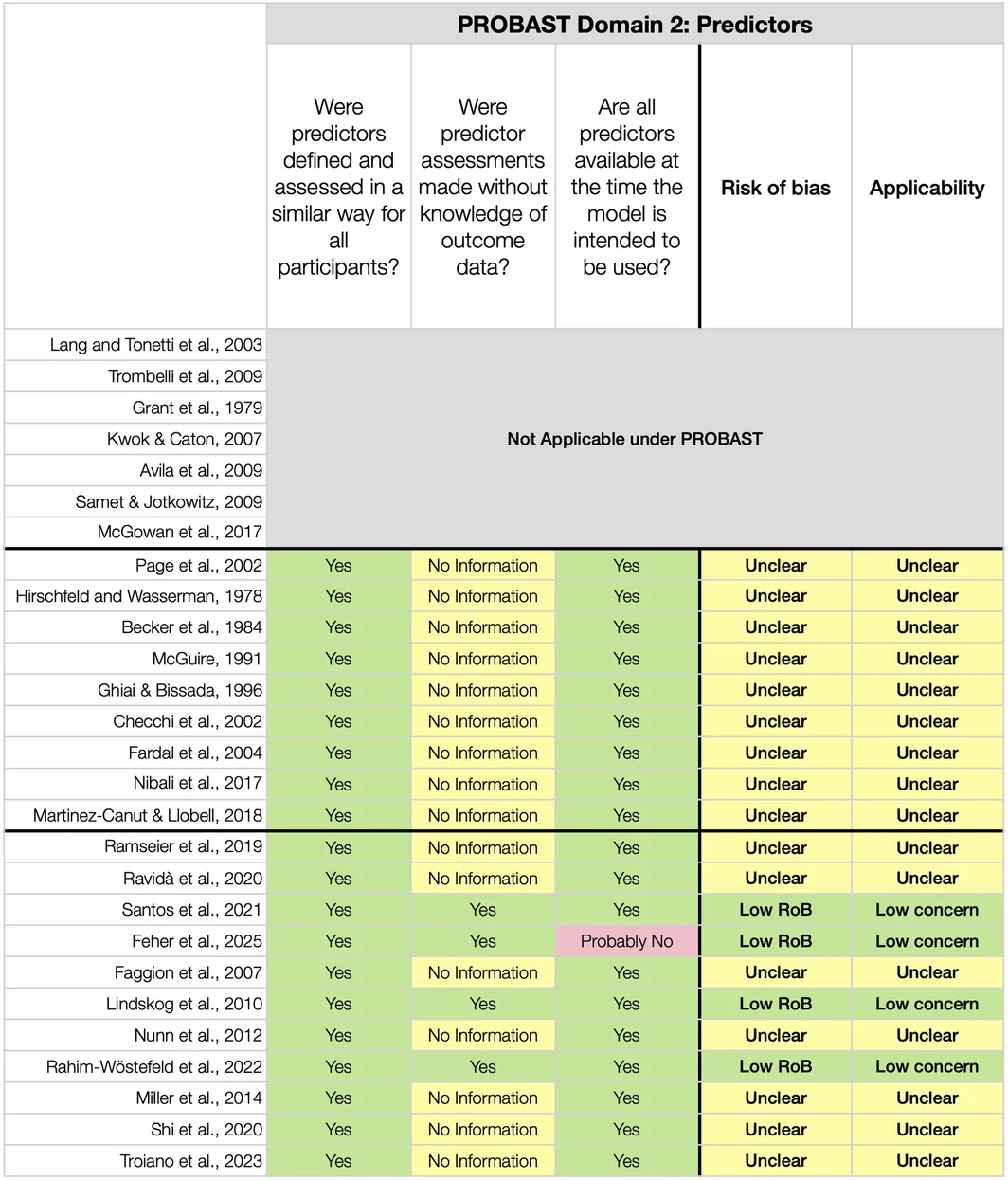
ROB assessment in the Predictors domain. Of 27 included studies, four studies showed ‘low’ risk, 16 were rated as ‘unclear’ and 7 were ‘not applicable’. Applicability concerns mirrored ROB ratings. A black horizontal line separates the three study categories: conceptual, category‐based, and data‐driven. Colour coding: green = low risk/concern, yellow = unclear risk/concern, red = high risk/concern, grey = not applicable.

Predictors such as pocket depth (PD), clinical attachment level (CAL), furcation involvement, tooth mobility and radiographic bone loss (RBL) were defined and assessed in a similar way for all participants across all studies (Hirschfeld and Wasserman 1978; Becker et al. 1984; Miller et al. 2014; McGuire 1991; Ghiai and Bissada 1996; Martinez‐Canut and Llobell 2018; Checchi et al. 2002; Page et al. 2002; Fardal et al. 2004; Nibali et al. 2017; Faggion et al. 2007; Lindskog et al. 2010; Nunn et al. 2012; Ramseier et al. 2019; Ravidà et al. 2020; Shi et al. 2020; Santos et al. 2021; Rahim‐Wöstefeld et al. 2022; Troiano et al. 2023; Feher et al. 2025) (Figure 3).

##### 3.3.2.2 Were Predictor Assessments Made Without Knowledge of Outcome Data?

Only 4 out of 20 studies ensured predictor assessment without knowledge of outcome data (data‐driven studies; Figure 3). Among these, two studies were prospective; therefore, the outcome was clearly not (yet) available to those assessing predictors (Lindskog et al. 2010; Rahim‐Wöstefeld et al. 2022), and two studies explicitly stated that outcome information was not used during predictor assessment (Santos et al. 2021; Feher et al. 2025). Category‐based and retrospective data‐driven studies (Hirschfeld and Wasserman 1978; Becker et al. 1984; Miller et al. 2014; McGuire 1991; Ghiai and Bissada 1996; Martinez‐Canut and Llobell 2018; Checchi et al. 2002; Page et al. 2002; Fardal et al. 2004; Nibali et al. 2017; Faggion et al. 2007; Nunn et al. 2012; Ramseier et al. 2019; Ravidà et al. 2020; Shi et al. 2020; Troiano et al. 2023) did not report whether predictor data collection was performed blinded to outcome information and were judged NI.

##### 3.3.2.3 Are All Predictors Available at the Time the Model Is Intended to Be Used?

Nineteen studies presented all predictors available at the time the model was intended to be used (Hirschfeld and Wasserman 1978; Becker et al. 1984; Miller et al. 2014; McGuire 1991; Ghiai and Bissada 1996; Martinez‐Canut and Llobell 2018; Checchi et al. 2002; Page et al. 2002; Fardal et al. 2004; Nibali et al. 2017; Faggion et al. 2007; Lindskog et al. 2010; Nunn et al. 2012; Ramseier et al. 2019; Ravidà et al. 2020; Shi et al. 2020; Santos et al. 2021; Rahim‐Wöstefeld et al. 2022; Troiano et al. 2023), such as PD, CAL, furcation involvement, mobility, RBL, smoking and diabetes (Figure 3). One study was rated PN (Feher et al. 2025), as the model incorporated the patient's microbial profile, a predictor that in routine practice would require additional laboratory processing and therefore is not readily available at the time of clinical decision making.

#### 3.3.3 Domain 3: Outcome

##### 3.3.3.1 Was the Outcome Determined Appropriately?

**FIGURE 4:**
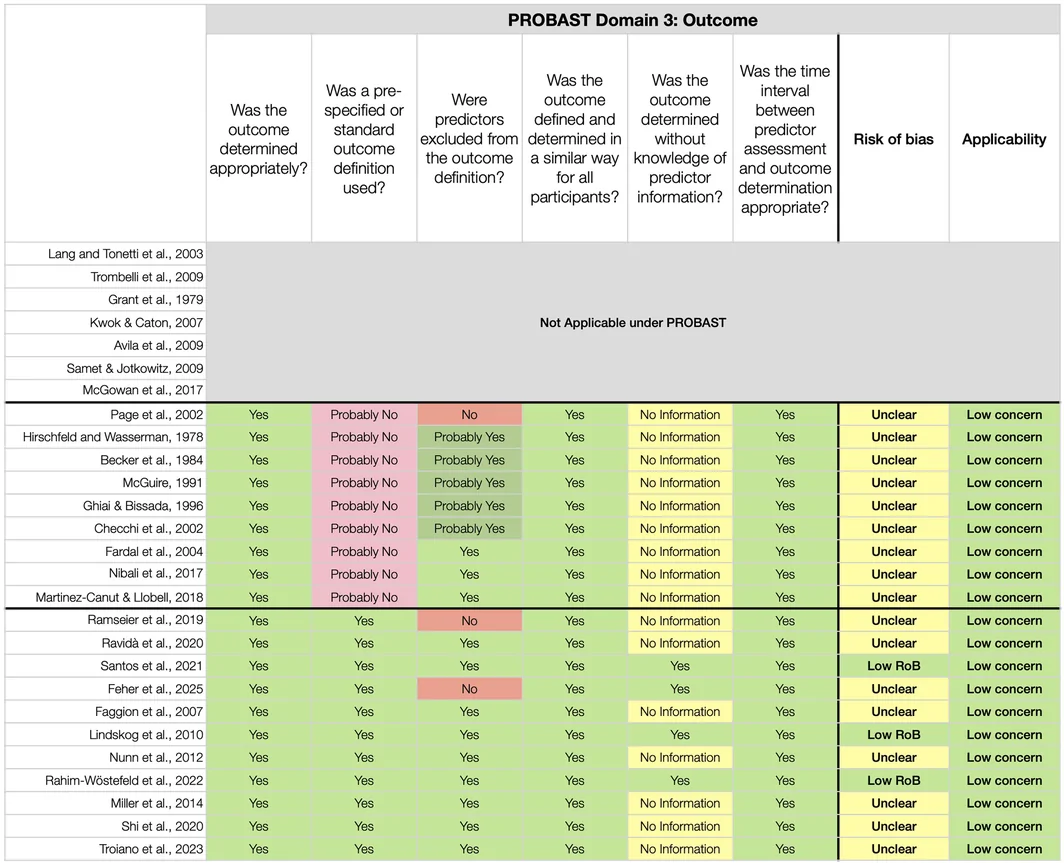
ROB assessment in the Outcomes domain. Of 27 included studies, three were judged as ‘low’ risk, 17 as ‘unclear’ and 7 as ‘not applicable’. Applicability concerns were judged as ‘low’ for category‐based and data‐driven studies and as ‘not applicable’ for the seven conceptual tools. A black horizontal line separates the three study categories: conceptual, category‐based, and data‐driven. Colour coding: green = low risk/concern, yellow = unclear risk/concern, red = high risk/concern, grey = not applicable.

Most studies determined outcomes appropriately, either by tooth loss documented through clinical/radiographic records, chart review, long‐term follow‐up (Hirschfeld and Wasserman 1978; Becker et al. 1984; Miller et al. 2014; McGuire 1991; Ghiai and Bissada 1996; Martinez‐Canut and Llobell 2018; Checchi et al. 2002; Fardal et al. 2004; Nibali et al. 2017; Faggion et al. 2007; Lindskog et al. 2010; Nunn et al. 2012; Ravidà et al. 2020; Shi et al. 2020; Santos et al. 2021; Rahim‐Wöstefeld et al. 2022; Troiano et al. 2023) or by disease progression/stability determined using established periodontal parameters such as PD, CAL and RBL (Page et al. 2002; Ramseier et al. 2019; Feher et al. 2025) (Figure 4).

##### 3.3.3.2 Was a Pre‐Specified or Standard Outcome Definition Used?

Outcome was considered standardised when its definition included a clear cause, defined time horizon and explicit assessment criteria. While all studies defined either tooth loss or disease progression/stability as endpoints, standardised outcome definitions were only infrequently reported. Among category‐based tools, outcomes were broadly defined and lacked either cause‐specific criteria or a clearly defined prediction horizon (e.g., tooth loss recorded during follow‐up without specifying whether it occurred within a predefined time frame) and were judged PN (Figure 4). Data‐driven models applied a standardised outcome definition.

##### 3.3.3.3 Were Predictors Excluded From the Outcome Definition?

In studies where tooth loss was the outcome, predictors were generally independent from the outcome definition (Hirschfeld and Wasserman 1978; Becker et al. 1984; Miller et al. 2014; McGuire 1991; Ghiai and Bissada 1996; Martinez‐Canut and Llobell 2018; Checchi et al. 2002; Fardal et al. 2004; Nibali et al. 2017; Faggion et al. 2007; Lindskog et al. 2010; Nunn et al. 2012; Ravidà et al. 2020; Shi et al. 2020; Santos et al. 2021; Rahim‐Wöstefeld et al. 2022; Troiano et al. 2023), with studies rated Y/PY (Figure 4). In studies where disease progression was the final outcome, none (Page et al. 2002; Ramseier et al. 2019; Feher et al. 2025) excluded predictors from the outcome definition; two studies included PD/residual pockets as predictors at baseline and also a part of the final outcome, while Page et al. (2002) used bone height and vertical bone lesions as predictors for RBL as an outcome.

##### 3.3.3.4 Was the Outcome Defined and Determined in a Similar Way for All Participants?

Across all studies, the outcome was defined and determined in a consistent manner for every participant/tooth/molar, typically documented through clinical standardised measures of periodontal progression or radiographic means (Figure 4).

##### 3.3.3.5 Was the Outcome Determined Without Knowledge of the Predictor?

Only a few studies explicitly stated that the outcome was determined without knowledge of predictor information (Lindskog et al. 2010; Santos et al. 2021; Rahim‐Wöstefeld et al. 2022; Feher et al. 2025) (Figure 4). Sixteen studies (Hirschfeld and Wasserman 1978; Becker et al. 1984; Miller et al. 2014; McGuire 1991; Ghiai and Bissada 1996; Martinez‐Canut and Llobell 2018; Checchi et al. 2002; Page et al. 2002; Fardal et al. 2004; Nibali et al. 2017; Faggion et al. 2007; Nunn et al. 2012; Ramseier et al. 2019; Ravidà et al. 2020; Shi et al. 2020; Troiano et al. 2023) were retrospective in design and did not report whether outcome data collection was performed blinded to predictor information, and were judged NI.

##### 3.3.3.6 Was the Time Interval Between Predictor Assessment and Outcome Determination Appropriate?

Across cohort studies, the interval between baseline predictor assessment and outcome determination was considered appropriate, ranging from 4 to 28 years (Hirschfeld and Wasserman 1978; Becker et al. 1984; Miller et al. 2014; McGuire 1991; Ghiai and Bissada 1996; Martinez‐Canut and Llobell 2018; Checchi et al. 2002; Page et al. 2002; Fardal et al. 2004; Nibali et al. 2017; Faggion et al. 2007; Lindskog et al. 2010; Nunn et al. 2012; Ramseier et al. 2019; Ravidà et al. 2020; Shi et al. 2020; Santos et al. 2021; Rahim‐Wöstefeld et al. 2022; Troiano et al. 2023) (Figure 4). Feher et al. (2025) had a 1‐year interval, which was still considered appropriate given the post‐therapy endpoints specified by the tool's intended use.

#### 3.3.4 Domain 4: Analysis

##### 3.3.4.1 Were There a Reasonable Number of Participants With the Outcome?

**FIGURE 5:**
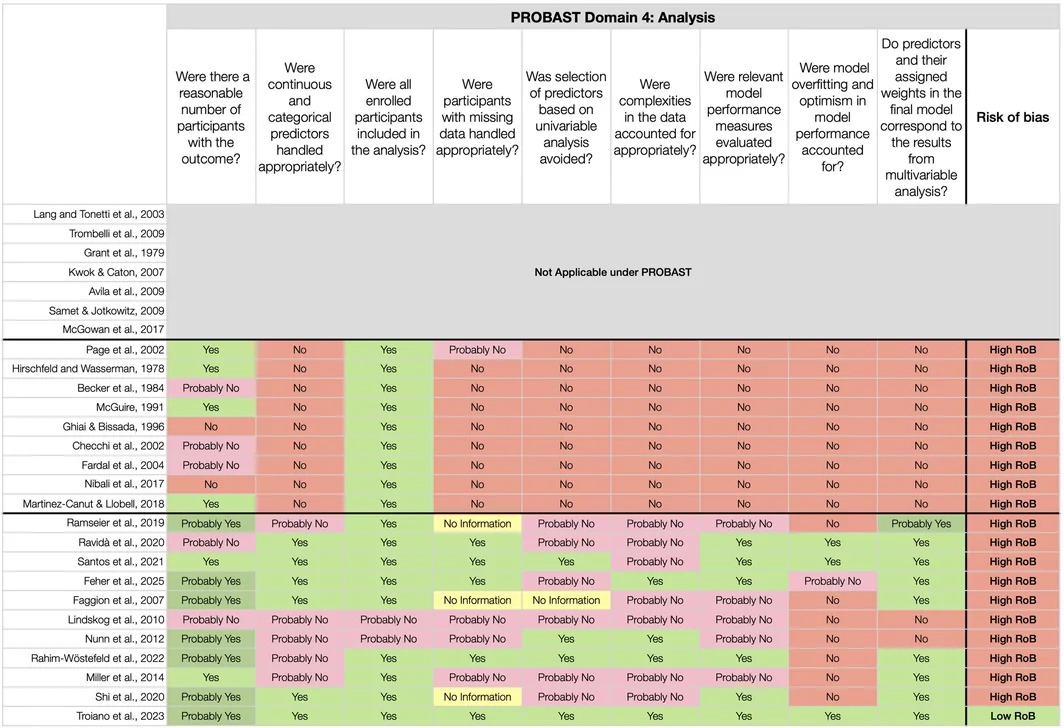
ROB assessments in the Analysis domain. Of 27 included studies, one was judged as ‘low’ risk, 19 as ‘high’ risk and 7 as ‘not applicable’. A black horizontal line separates the three study categories: conceptual, category‐based, and data‐driven. Colour coding: green = low risk/concern, yellow = unclear risk/concern, red = high risk/concern, grey = not applicable.

Among data‐driven models, most fulfilled the recommended thresholds for the number of participants with the outcome (Figure 5). Across nine studies where tooth loss served as the outcome, two reported adequate event‐per‐variable (EPV) ratios ≥ 20 (Miller et al. 2014; Santos et al. 2021) (range 20.6–29.5). Five studies achieved intermediate EPV (5–19) and were rated PY: 6 (Nunn et al. 2012), 8.7 (Shi et al. 2020), 8.6 (Troiano et al. 2023), 15.1 (Rahim‐Wöstefeld et al. 2022) and 19.2 (Faggion et al. 2007). Two studies reported fewer events per parameter (EPV 3.3 (Lindskog et al. 2010) and 4.2 (Ravidà et al. 2020)) and were rated PN, raising concerns about potential overfitting. Across two studies, where disease progression served as the outcome, one (Ramseier et al. 2019) used residual PD to define stability/progression and reported an EPV of 15.3, which was rated PY. The other (Feher et al. 2025) developed their model in 414 patients, of whom 175 achieved stability after 1 year, corresponding to an EPV of 9.6 across 18 predictors, rated PY. Among category‐based studies, only a few met the recommended thresholds for the number of participants with events. In three investigations where tooth loss was the outcome (Hirschfeld and Wasserman 1978; McGuire 1991; Martinez‐Canut and Llobell 2018), the number of teeth lost was > 100. Three studies (Becker et al. 1984; Checchi et al. 2002; Fardal et al. 2004) reported teeth loss from 50 to 67 and were rated PN, while two studies (Ghiai and Bissada 1996; Nibali et al. 2017) reported 3145 teeth lost and were rated N. The study (Page et al. 2002) that defined the outcome as disease progression determined by RBL documented ≥ 100 events, fulfilling the validation criterion.

##### 3.3.4.2 Were Continuous and Categorical Predictors Handled Appropriately?

Among data‐driven studies, handling of predictors showed substantial heterogeneity (Figure 5). Six studies (Faggion et al. 2007; Ravidà et al. 2020; Shi et al. 2020; Santos et al. 2021; Troiano et al. 2023; Feher et al. 2025) retained continuous predictors in their statistical or machine‐learning models and in some cases assessed potential non‐linear effects and thus appropriately handled continuous and categorical predictors. Five studies (Miller et al. 2014; Lindskog et al. 2010; Nunn et al. 2012; Ramseier et al. 2019; Rahim‐Wöstefeld et al. 2022) relied on tree‐based methods or threshold‐driven scoring systems and categorised continuous predictors, thus rated PN. Across category‐based studies, predictors were uniformly categorised using predefined thresholds. Variables such as PD, CAL, mobility and furcation involvement were consistently transformed into categorical groups. While this approach was clinically intuitive and applied consistently, it does not align with current methodological recommendations for retaining continuous variables. Accordingly, category‐based studies were judged N (Hirschfeld and Wasserman 1978; Becker et al. 1984; McGuire 1991; Ghiai and Bissada 1996; Martinez‐Canut and Llobell 2018; Checchi et al. 2002; Page et al. 2002; Fardal et al. 2004; Nibali et al. 2017).

##### 3.3.4.3 Were All Enrolled Participants Included in the Analysis?

Most data‐driven studies analysed all available cases, ensuring consistency between enrolment and analysis (Miller et al. 2014; Faggion et al. 2007; Ramseier et al. 2019; Ravidà et al. 2020; Shi et al. 2020; Santos et al. 2021; Rahim‐Wöstefeld et al. 2022; Troiano et al. 2023; Feher et al. 2025). However, two studies (Lindskog et al. 2010; Nunn et al. 2012) excluded participants with incomplete data without reporting imputation or handling methods and were judged PN. Across category‐based tools, all enrolled participants were included in the final analyses, with no evidence of post hoc exclusions (Figure 5). Importantly, these participants were not used for model development but to evaluate predefined prognostic categories.

##### 3.3.4.4 Were Participants With Missing Data Handled Appropriately?

Some data‐driven studies did not report appropriate methods for handling missing data, generally excluding participants with incomplete records (Miller et al. 2014; Lindskog et al. 2010; Nunn et al. 2012) and considered PN/N (Figure 5). Recent models (Ravidà et al. 2020; Santos et al. 2021; Rahim‐Wöstefeld et al. 2022; Troiano et al. 2023; Feher et al. 2025) applied explicit methods to handle missing predictors, including imputation strategies, and were rated Y. Three studies provided insufficient details and were judged NI (Faggion et al. 2007; Ramseier et al. 2019; Shi et al. 2020). Complete‐case analysis was also common among category‐based studies (Hirschfeld and Wasserman 1978; Becker et al. 1984; McGuire 1991; Ghiai and Bissada 1996; Martinez‐Canut and Llobell 2018; Checchi et al. 2002; Fardal et al. 2004; Nibali et al. 2017). One study (Page et al. 2002) provided some reporting of exclusions related to incomplete data, although no formal imputation methods were applied and was thus judged PN.

##### 3.3.4.5 Was the Selection of Predictors Based on Univariable Analysis Avoided?

Among data‐driven models, four studies (Nunn et al. 2012; Santos et al. 2021; Rahim‐Wöstefeld et al. 2022; Troiano et al. 2023) avoided univariable screening, either by using pre‐specified predictor sets or algorithms that embed selection without *p*‐value filtering (CART/GLMM‐tree, penalised or ML pipelines), and were rated Y (Figure 5). Six studies (Miller et al. 2014; Lindskog et al. 2010; Ramseier et al. 2019; Ravidà et al. 2020; Shi et al. 2020; Feher et al. 2025) selected predictors via univariable screening or stepwise procedures and were rated PN. One study (Faggion et al. 2007) did not report their selection strategy sufficiently and was judged NI. Category‐based studies (Hirschfeld and Wasserman 1978; Becker et al. 1984; McGuire 1991; Checchi et al. 2002; Page et al. 2002; Fardal et al. 2004; Nibali et al. 2017) did not involve multivariable modelling and relied on predefined or clinically derived predictor selection, and therefore were judged N.

##### 3.3.4.6 Were Complexities in the Data (e.g., Non‐Linear Relationships) Handled Appropriately?

Across nine data‐driven studies where tooth loss served as the outcome, three (Nunn et al. 2012; Rahim‐Wöstefeld et al. 2022; Troiano et al. 2023) applied time‐to‐event methods that explicitly accounted for censoring, and were rated Y (Figure 5). The other tooth loss studies (Miller et al. 2014; Faggion et al. 2007; Lindskog et al. 2010; Ravidà et al. 2020; Shi et al. 2020; Santos et al. 2021) relied on binary endpoints without time‐to‐event adjustment and were rated PN. Across two studies where disease progression served as the outcome, the first (Ramseier et al. 2019) defined stability/progression by residual PD without time‐to‐event analysis and was rated PN, whereas the second (Feher et al. 2025) employed Cox proportional hazards models and was judged Y. Category‐based studies reported proportions or descriptive survival but did not consistently apply time‐to‐event methods or account for censoring; nor did they involve formal model development, and were judged N.

##### 3.3.4.7 Were the Relevant Model Performance Measures Evaluated Appropriately?

Six data‐driven studies (Ravidà et al. 2020; Shi et al. 2020; Santos et al. 2021; Rahim‐Wöstefeld et al. 2022; Troiano et al. 2023; Feher et al. 2025) reported both discrimination and calibration, meeting recommended standards and were rated Y (Figure 5). The remaining (Miller et al. 2014; Faggion et al. 2007; Lindskog et al. 2010; Nunn et al. 2012; Ramseier et al. 2019) restricted evaluation to discrimination metrics such as area under the curve (AUC), sensitivity/specificity or cross‐validated error, without calibration, and were rated PN. Category‐based tools (Hirschfeld and Wasserman 1978; Becker et al. 1984; McGuire 1991; Ghiai and Bissada 1996; Martinez‐Canut and Llobell 2018; Checchi et al. 2002; Page et al. 2002; Fardal et al. 2004; Nibali et al. 2017) typically reported tooth survival rates, hazard ratios or Kaplan–Meier survival curves but did not involve formal model development or performance measures; therefore, they were judged N.

##### 3.3.4.8 Were Model Overfitting and Optimism in Model Performance Accounted for?

Within data‐driven models, three implemented appropriate methods to mitigate overfitting and optimism, being judged Y (Figure 5). Troiano et al. (2023) applied cross‐validation during model development and additionally reported external validation; Santos et al. (2021) performed a non‐random, split‐sample validation approach, while Ravidà et al. (2020) applied internal validation through random split‐sample testing and calibration slope adjustment. One study (Feher et al. 2025) used only a split‐sample approach and was judged PN. The remaining data‐driven studies (Miller et al. 2014; Faggion et al. 2007; Lindskog et al. 2010; Nunn et al. 2012; Ramseier et al. 2019; Shi et al. 2020; Rahim‐Wöstefeld et al. 2022) did not apply any form of internal validation and were rated N. Among category‐based studies, none implemented internal validation procedures or formal model development, and were rated N.

##### 3.3.4.9 Do Predictors and Their Assigned Weights in the Final Model Correspond to the Results From the Reported Multivariable Analysis?

Within data‐driven models, nine studies (Miller et al. 2014; Faggion et al. 2007; Ramseier et al. 2019; Ravidà et al. 2020; Shi et al. 2020; Santos et al. 2021; Rahim‐Wöstefeld et al. 2022; Troiano et al. 2023; Feher et al. 2025) provided regression‐ or machine‐learning‐based models in which predictors and their assigned weights corresponded appropriately to the results of multivariable analyses, and thus were judged Y (Figure 5). Two studies (Lindskog et al. 2010; Nunn et al. 2012) presented multivariable analyses but applied scoring transformations that did not directly correspond to the reported coefficients, and were thus rated N. Category‐based studies did not develop regression‐based multivariable models despite using empirical data; they relied solely on predefined prognostic categories and did not assign predictor weights based on multivariable analysis, and therefore were rated N.

#### 3.3.5 Overall ROB Assessment Across Studies

**FIGURE 6:**
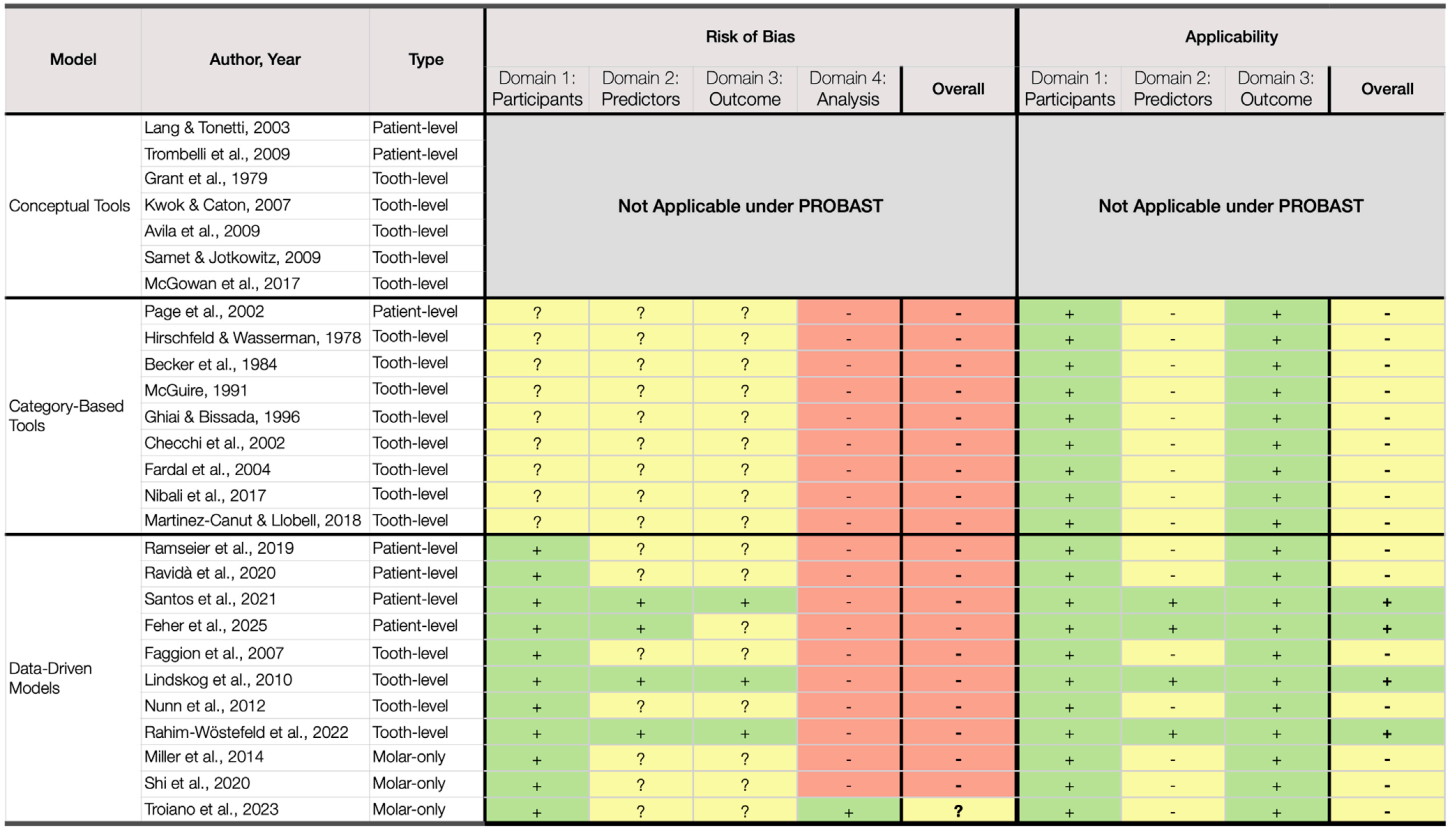
Overall risk of bias (ROB) and applicability assessments across the 27 included studies. The table summarises ROB in each PROBAST domain (Participants, Predictors, Outcomes and Analysis) and applicability concerns across corresponding domains. Overall ROB was considered ‘not applicable’ for conceptual tools, ‘high’ for category‐based tools and predominantly ‘high’ for data‐driven models, with one study classified as ‘unclear’. Applicability showed a more heterogeneous distribution: conceptual models were ‘not applicable’ across applicability domains; category‐based models were predominantly unclear, primarily reflecting heterogeneity in predictor specification across schemes; data‐driven models demonstrated a mixed profile, including both low and unclear concern ratings. Colour coding: green = low risk/concern, yellow = unclear risk/concern, red = high risk/concern, grey = not applicable.

Data‐driven models demonstrated high ROB across multiple domains, with one study (Troiano et al. 2023) rated as unclear ROB due to insufficient reporting (Figures 6 and S2). Category‐based studies were included in PROBAST but were not formal prediction models; their appraisal reflected outcome‐based evaluation of predefined prognostic classifications rather than prediction model development or performance assessment.

### 3.4 Applicability: PROBAST Domains Assessment

See Supporting Information.

### 3.5 Reporting Standards

Studies before 2014 did not explicitly mention reporting standards (Hirschfeld and Wasserman 1978; Becker et al. 1984; Miller et al. 2014; Grant et al. 1979; McGuire 1991; Ghiai and Bissada 1996; Checchi et al. 2002; Page et al. 2002; Fardal et al. 2004; Lindskog et al. 2010; Nunn et al. 2012). After 2014, four studies reported adherence to TRIPOD (Ravidà et al. 2020; Santos et al. 2021) or TRIPOD+AI (Troiano et al. 2023; Feher et al. 2025). No mention of reporting standards was found in six studies (McGowan et al. 2017; Martinez‐Canut and Llobell 2018; Nibali et al. 2017; Ramseier et al. 2019; Shi et al. 2020; Rahim‐Wöstefeld et al. 2022). ROB distribution by TRIPOD timing is shown in Figure S3.

### 3.6 Methodological Improvements Across Study Designs

The 27 studies included in this review reflect distinct methodological approaches spanning the evolution of prognostic modelling in periodontology. Conceptual tools (*n* = 7; 1979–2010) proposed non‐empirical frameworks without quantitative risk estimation. While influential in clinical decision making, their limitations reflect evaluability gaps rather than predictive bias. Category‐based systems (*n* = 9; 1980–2015) advanced prognostic thinking through structured clinical classifications applied to patient cohorts. However, they uniformly categorised continuous predictors and lacked formal validation, reflecting pre‐contemporary standards for quantitative modelling and calibration assessment. Data‐driven models (*n* = 11; 2010+) demonstrate progressive methodological refinement. Earlier models (2010–2018) lacked consistent validation protocols, whereas recent studies (2020+) more frequently report internal validation (*n* = 3), discrimination and calibration metrics (*n* = 6) and external validation (*n* = 2), aligning with contemporary standards. Across all designs, predictor definitions (PD, CAL, mobility, RBL) were consistently operationalised and clinically applicable. Outcomes (tooth loss, disease progression) were clinically meaningful and standardised within design types. Persistent limitations included reliance on case deletion for missing data, underuse of time‐to‐event methods and categorisation of continuous variables across conceptual, category‐based and earlier data‐driven approaches. Calibration assessment and external validation remained largely confined to recent data‐driven models. This classification illustrates the progression of prognostic thinking from non‐empirical frameworks (conceptual), through structured categorical systems (category‐based), to quantitative prediction modelling (data‐driven). Conceptual and category‐based approaches remain historically and clinically informative, while data‐driven models represent the current methodological standard.

## 4 Discussion

This systematic review appraised the methodological quality and historical evolution of 27 prognostic models for periodontitis developed between 1979 and 2025. Studies were stratified into conceptual, category‐based and data‐driven designs to capture methodological evolution. Across these approaches, progressive improvements were observed in the specification of predicted outcomes, definition of prediction horizons and format of risk estimation. Prognostic methods evolved from qualitative risk categories and expert‐based classifications towards quantitative, individualised probability estimates generated through multivariable statistical and machine‐learning models.

ROB was assessed for category‐based and data‐driven models and, although overall ratings remained predominantly high, domain‐level assessments revealed important methodological differences between study designs. Data‐driven models showed high ROB concentrated in the Analysis domain, driven by gaps in missing data handling, validation and calibration assessment. Category‐based tools, while evaluated by PROBAST, reflected design limitations (absence of statistical modelling) rather than bias in predicted performance. Because PROBAST is intentionally stringent, a single high‐risk domain is sufficient to classify an entire study as high risk. Consequently, several studies with predominantly positive signalling‐question responses remained classified as high ROB due to deficiencies concentrated in the Analysis domain.

This review was not designed to establish which prognostic tool performs best. That question has already been addressed in comparative validation studies (Saleh et al. 2021; Saleh et al. 2022; Saleh et al. 2023; Petsos et al. 2020). Instead, the present study focused on whether these models are being developed and reported in alignment with contemporary methodological standards. Our findings build upon and complement prior reviews (Chow et al. 2024; Du et al. 2018), which applied TRIPOD, CHARMS and PROBAST to periodontal prognostic approaches. Unlike previous reviews, which largely focused on validation studies or combined prediction/prognostic outcomes, the present analysis included only original model‐development articles and compared their methodological rigour across three historically distinct design types. In addition, a structured synthesis of all models across key dimensions (e.g., predicted outcome, prediction time, model output and clinical application) has been presented, enabling direct comparison of how prognostic models are defined and used. This level of contextualisation has not been previously reported in the literature.

Despite the availability of CHARMS, TRIPOD and PROBAST for more than a decade, ROB ratings remain consistently high, and adherence to key methodological requirements is inconsistent. This contrasts with other areas of clinical research, where adherence to PRISMA (Moher et al. 2015), CONSORT (Hopewell et al. 2025) or STROBE (von Elm et al. 2014) has improved transparency and reproducibility. The fact that many studies continue to neglect fundamental reporting standards highlights the need for better awareness, education and enforcement of these frameworks. This limited impact may reflect not only inconsistent enforcement of reporting guidelines but also limited familiarity with prediction modelling methods among dental researchers and continued reliance on traditional, qualitative prognostic models.

The tripartite classification introduced in this review provides a methodological structure not previously applied in periodontology. By distinguishing conceptual frameworks, category‐based systems and data‐driven models, this approach clarifies how predictor definitions, outcome formulations and analytical standards evolved across different eras of prognostic model development. Beyond assessing methodological quality, the review maps the historical progression of periodontal prognostic models, highlighting consistent strengths (e.g., stability of core predictor definitions) and persistent weaknesses (e.g., scarce calibration, limited external validation, dichotomisation of continuous variables). Addressing these gaps will require methodological training for researchers and stronger editorial enforcement of reporting standards. Interdisciplinary collaboration with statisticians and data scientists is also critical to strengthening modelling practices. Evaluation of practices before and after the introduction of TRIPOD/PROBAST further demonstrates the limited adoption of these frameworks and underscores the need for methodological benchmarks to advance clinically reliable prognostic modelling.

This review has limitations. PROBAST was applied only to category‐based and data‐driven models. Conceptual tools were not evaluable under PROBAST because they lack empirical data, outcome assessment and formal model development, although they remain historically important in the evolution of periodontal prognostication. Category‐based tools may be disadvantaged when evaluated using PROBAST because they were not designed as modern prediction models and therefore lack features such as formal model fitting, calibration assessment and validation procedures. Consequently, high ROB ratings should be interpreted as reflecting differences in methodological standards rather than evidence of poor clinical utility. Finally, most included category‐based and data‐driven studies were retrospective cohorts. While PROBAST recognises that retrospective datasets may carry a higher ROB due to non‐prespecified measurement protocols, missing data handling and blinding procedures, such designs are common in periodontal prognosis research because clinically meaningful outcomes such as tooth loss and disease progression often require follow‐up periods of 10 years or longer. Where reporting of these methodological aspects was incomplete, our ability to fully assess bias was limited, and several studies received unclear rather than low‐risk ratings on this domain.

Future research must prioritise adherence to TRIPOD/TRIPOD‐AI, prospective data collection where feasible and the adoption of advanced analytical strategies, and ensure transparent reporting. Only through such methodological refinement will prognostic models in periodontology meaningfully contribute to precision dentistry.

## 5 Conclusions

Prognostic models in periodontology reflect distinct methodological approaches shaped by historical evolution. Conceptual frameworks are not evaluable under PROBAST and represent qualitative contributions to clinical thinking. Category‐based systems employed predefined classifications that do not align with contemporary standards for quantitative modelling and formal validation. Data‐driven models demonstrate progressive refinement, with recent studies increasingly reporting internal validation, calibration metrics and external validation; however, improvements remain sporadic rather than systematic. Persistent gaps in missing data handling, time‐to‐event methods and transparent reporting require sustained attention. Future prognostic research must prioritise adherence to TRIPOD‐AI, prospective data collection with prespecified protocols and structured validation strategies. Only through systematic adoption of contemporary standards will prognostic models meaningfully contribute to precision dentistry.

## Author Contributions

M.K., D.R.D., G.T. and A.R. contributed to the study conception and design, data collection, analysis and interpretation, as well as manuscript drafting and critical revision. I.A. and D.J. contributed to data collection. S.B. and M.S. contributed to data interpretation, manuscript drafting and critical revision. All the authors gave their final approval of the version to be published and agreed to be accountable for all aspects of the work.

## Funding

The authors have nothing to report.

## Conflicts of Interest

The authors declare no conflicts of interest.

## Supporting information


**Table S1:** Search strategy and keywords in each database.
**Table S2:** Eleven domains of the CHARMS checklist were systematically completed for each study. The PROBAST checklist includes four key domains (Participants, Predictors, Outcome, Analysis) with a total of 20 questions to answer.
**Figure S1:** PRISMA flowchart.
**Table S3:** Comparison of how primary studies were classified across the current review (Karaban et al.), and previous ones (Chow et al. 2024; Du et al. 2018). The present review included only studies that developed prognostic tools. Studies focused exclusively on tooth‐loss prediction, diagnostic tools or validation/modification of previously published models were not selected. Green cells indicate studies included in a given review, while red cells indicate exclusion. Studies published after the search periods of Chow et al. (2024) and Du et al. (2018) are shown on the right for reference.
**Table S4:** Comprehensive overview of participants characteristics, including number, male/female numbers, age and smokers.
**Table S5:** Comprehensive overview of model characteristics, including modelling method, sample size, number of predictors, selection of final predictors, missing data handling, type of validation and performance measures.
**Figure S2:** Frequency distribution of responses (‘Yes’, ‘No’, ‘Probably Yes’, ‘Probably No’ and ‘No Information’) for each PROBAST question across 20 studies in which PROBAST was applied.
**Figure S3:** Proportions of studies with low (green), unclear (yellow), high (red) and not applicable (grey) ROB across the four domains (Participants, Predictors, Outcomes, Analysis) before and after 2014.

## Data Availability

Data is available upon request to the corresponding author.
