# Supplementary material for: Methodological Quality of Prognostic Models for Periodontitis: A Systematic Review and Critical Appraisal
Source: J Clin Periodontol. 2026 Aug 3;53(10):1513–28. doi: 10.1111/jcpe.70180 (PMC13581475; doi:10.1111/jcpe.70180)
Supplement: Supplementary file 1 [file JCPE-53-1513-s001.docx]

**Methodological Quality of Prognostic Models for Periodontitis: A Systematic Review and Critical Appraisal**

Maryia Karaban; Debora R. Dias; Giuseppe Troiano; Sahar Baniameri; Ishan Ahuja; Divya Joseph; Matteo Serroni; Andrea Ravidà

**SUPPLEMENTARY MATERIALS**

[2. Material and methods:	3](#_Toc232780385)

[2.4. Search Strategy:	3](#_Toc232780386)

[*Supplementary Table 1.* Search strategy and keywords in each database:	3](#_Toc232780387)

[2.5. Data collection process	6](#_Toc232780388)

[*Supplementary Table 2.* Eleven domains of the CHARMS checklist were systematically completed for each study. The PROBAST checklist includes four key domains (Participants, Predictors, Outcome, Analysis) with a total of 20 questions to answer.	6](#_Toc232780389)

[2.6. Risk of bias (ROB) assessment	11](#_Toc232780391)

[2.7. Statistical Analysis	12](#_Toc232780392)

[3. Results:	12](#_Toc232780393)

[3.1. Study selection and characteristics	12](#_Toc232780394)

[Selection	12](#_Toc232780395)

[*Figure S1.* PRISMA flowchart	14](#_Toc232780396)

[*Supplementary Table 3.* Comparison of how primary studies were classified across the current review (Karaban et al.), and previous ones (Chow, Tay, & Nascimento, 2024; Du, Bo, Kapellas, & Peres, 2018). The present review included only studies that developed prognostic tools. Studies focused exclusively on tooth-loss prediction, diagnostic tools, or validation/modification of previously published models were not selected. Green cells indicate studies included in a given review, while red cells indicate exclusion. Studies published after the search periods of Chow et al. and Du et al. are shown on the right for reference.	16](#_Toc232780397)

[Characteristics of included studies	19](#_Toc232780398)

[*Supplementary Table 4.* Comprehensive overview of participants characteristics, including number, male/female numbers, age, smokers.	21](#_Toc232780399)

[*Supplementary Table 5.* Comprehensive overview of model characteristics, including modeling method, sample size, number of predictors, selection of final predictors, missing data handling, type of validation, performance measures.	26](#_Toc232780400)

[*Figure S2.* Frequency distribution of responses (“Yes,” “No,” “Probably Yes”, “Probably No”, and “No Information”) for each PROBAST question across 20 studies in which PROBAST was applied.	37](#_Toc232780401)

[3.2. Summary of included studies	38](#_Toc232780402)

[3.4. Applicability Assessment	41](#_Toc232780403)

[3.4.1. Domain 1: Participants	41](#_Toc232780404)

[3.4.2. Domain 2: Predictors	41](#_Toc232780405)

[3.4.3. Domain 3: Outcome	42](#_Toc232780406)

[3.4.4. Overall Applicability	42](#_Toc232780407)

[3.5. Reporting Standards	42](#_Toc232780408)

[3.5.1. Methodological Improvements After TRIPOD Introduction: A Comparative Risk Analysis	42](#_Toc232780409)

[*Figure S3.* Proportions of studies with low (green), unclear (yellow), high (red), and not applicable (gray) ROB across the four domains (Participants, Predictors, Outcomes, Analysis) before and after 2014.	44](#_Toc232780410)

[References	45](#_Toc232780411)

# 2. Material and methods:

## 2.4. Search Strategy:

Studies were identified using Medline/PubMed, Embase, and Cochrane. The search process was completed in May 2025. The search strategy combined MeSH terms and free-text keywords with Boolean operators as shown in Supplementary Table 1. Additionally, reference lists of included studies were manually reviewed to identify further relevant studies. Three reviewers (M.K./I.A./D.J.) independently selected studies using Rayyan software. A fourth reviewer (D.R.D.) resolved discrepancies.

## Supplementary Table 1. Search strategy and keywords in each database:

| Database | Keywords |  |
| --- | --- | --- |
| Pubmed | (((("periodontitis") OR ("periodontal disease"))  AND  ((((((((((((((((((("periodontal risk assessment") OR ("prognostic systems")) OR ("risk score")) OR ("model stratification")) OR ("prognostic models")) OR ("risk factor")) OR ("risk assessment")) OR ("supportive periodontal therapy")) OR ("supportive care")) OR (maintenance)) OR ("risk management")) OR (risk evaluation and mitigation)) OR (validation)) OR (forecasting)) OR ("predictive modelling")) OR ("scoring index")) OR ("tooth prognosis")) OR ("prognostic factor")) OR ("risk calculation")) OR ("risk estimation")))  AND  (((((((("alveolar bone loss") OR ("tooth loss")) OR ("disease progression")) OR ("clinical attachment loss"))) OR ("tooth survival"))))  NOT  ((review) OR (cancer)) | 1345 |
| Cochrane | #1 ("periodontitis"):ti,ab,kw OR ("periodontal disease"):ti,ab,kw  #2 (periodontal risk assessment):ti,ab,kw OR (prognostic system):ti,ab,kw OR ("risk score"):ti,ab,kw OR (model stratification):ti,ab,kw OR (prognostic models):ti,ab,kw  #3 ("risk-factor"):ti,ab,kw OR ("risk assessment"):ti,ab,kw OR (risk evaluation):ti,ab,kw OR (validation):ti,ab,kw OR (forecasting):ti,ab,kw  #4 (predictive modelling):ti,ab,kw OR (scoring index):ti,ab,kw OR (tooth prognosis):ti,ab,kw OR ("prognostic factor"):ti,ab,kw OR (risk calculation):ti,ab,kw  #5 (risk estimation):ti,ab,kw  #6 #2 OR #3 OR #4 OR #5  #7 ("alveolar bone loss"):ti,ab,kw OR (tooth loss):ti,ab,kw OR (disease progression):ti,ab,kw OR ("clinical attachment loss"):ti,ab,kw OR (tooth survival):ti,ab,kw  #8 #1 AND #6 AND #7 | 187 |
| Embase | ('periodontitis'exp OR 'paradontitis' OR 'parodontitis' OR 'peridontitis' OR 'periodontitis' OR 'periodontal disease'exp OR 'dental loss' OR 'peridontal disease' OR 'peridontal tissue disease' OR 'peridontium disease' OR 'periodontal disease' OR 'periodontal diseases' OR 'periodontal infection' OR 'periodontium disease' OR 'tooth loss' OR 'tooth prognosis') AND ('prognostic assessment'/exp OR 'prognostic assessment' OR 'prognostic score/exp OR 'prognostic stratification'/exp OR 'risk assessment'/exp OR 'risk adjustment' OR 'risk analysis' OR 'risk assessment' OR 'risk evaluation' OR 'risk score'exp OR 'risk factor'exp OR 'relative risk' OR 'risk factor' OR 'risk factors' OR 'periodontal risk assessment' OR 'supportive periodontal therapy'/exp OR 'risk management/exp OR 'risk management' OR 'risk planning and management' OR 'validation'/exp OR 'forecasting'/exp OR 'fore-casting' OR 'forecasting' OR 'predictive model'/exp OR 'forecast model' OR 'forecast modeling' OR 'forecast modelling' OR 'forecast simulation' OR 'forecasting model' OR 'prediction model' OR 'predictive model' OR 'predictive modeling' OR 'predictive modelling' OR 'predictive simulation' OR 'risk calculator/exp OR 'risk estimation'/exp)  AND  ('alveolar bone loss'exp OR 'alveolar bone loss' OR 'periodontal bone loss' OR 'disease exacerbation'/exp OR 'disease progression' OR 'clinical attachment level'/exp OR 'clinical attachment loss' OR 'tooth survival')  AND  ('randomized controlled trial'/exp OR 'controlled trial, randomized' OR 'randomised controlled study' OR 'randomised controlled trial' OR 'randomized controlled study' OR 'randomized controlled trial' OR 'trial, randomized controlled' OR 'cohort analysis/exp OR 'analysis, cohort' OR 'cohort analysis' OR 'cohort studies' OR 'cohort study')  NOT  'review' | 225 |

## 2.5. Data collection process

Data from each selected study were extracted using the CHARMS checklist. Eleven domains were completed: Source of Data, Participants, Outcome(s) to be Predicted, Candidate Predictors, Sample Size, Missing Data, Model Development, Model Performance, Model Evaluation, Results, and Interpretation and Discussion. Domains of the CHARMS and PROBAST were described in detail in Supplementary Table 2.

## Supplementary Table 2. Eleven domains of the CHARMS checklist were systematically completed for each study. The PROBAST checklist includes four key domains (Participants, Predictors, Outcome, Analysis) with a total of 20 questions to answer.

| CHARMS | | PROBAST | |
| --- | --- | --- | --- |
| 1. Source of Data | 1.1 Source of data (e.g., cohort, case-control, randomized trial participants, or registry data) | 1. Participants | 1.1 Were appropriate data sources used (e.g., cohort, RCT, or nested case-control study data)? |
| 2. Participants | 2.1 Participant eligibility and recruitment method (e.g., consecutive participants, location, number of centers, setting, inclusion, and exclusion criteria) |  | 1.2 Were all the inclusions and exclusions of participants appropriate? |
| 3. Outcome(s) to be Predicted | 3.1 Definition and method for measurement of outcome | 2. Predictors | 2.1 Were predictors defined and assessed in a similar way for all participants? |
|  | 3.2 Was the same outcome definition (and method for measurement) used in all patients? |  | 2.2 Were predictor assessments made without knowledge of outcome data? |
|  | 3.3 Type of outcome (e.g., single or combined endpoints) |  | 2.3 Are all predictors available at the time the model is intended to be used? |
|  | 3.4 Was the outcome assessed without knowledge of the candidate predictors (i.e., blinded)? | 3. Outcome | 3.1 Was the outcome determined appropriately? |
|  | 3.5 Were candidate predictors part of the outcome (e.g., in panel or consensus diagnosis)? |  | 3.2 Was a pre-specified or standard outcome definition used? |
|  | 3.6 Time of outcome occurrence or summary of duration of follow-up |  | 3.3 Were predictors excluded from the outcome definition? |
| 4. Candidate Predictors (or Index Tests) | 4.1 Number and type of predictors (e.g., demographics, patient history, physical examination, additional testing, disease characteristics) |  | 3.4 Was the outcome defined and determined in a similar way for all participants? |
|  | 4.2 Definition and method for measurement of candidate predictors |  | 3.5 Was the outcome determined without knowledge of predictor information? |
|  | 4.3 Timing of predictor measurement (e.g., at patient presentation, at diagnosis, at treatment initiation) |  | 3.6 Was the time interval between predictor assessment and outcome determination appropriate? |
|  | 4.4 Were predictors assessed blinded for outcome, and for each other (if relevant)? | 4. Analysis | 4.1 Were there a reasonable number of participants with the outcome? |
|  | 4.5 Handling of predictors in the modelling (e.g., continuous, linear, non-linear |  | 4.2 Were continuous and categorical predictors handled appropriately? |
| 5. Sample Size | 5.1 Number of participants and number of outcomes/events |  | 4.3 Were all enrolled participants included in the analysis? |
|  | 5.2 Number of outcomes/events in relation to the number of candidate predictors (Events Per Variable) |  | 4.4 Were participants with missing data handled appropriately? |
| 6. Missing Data | 6.1 Number of participants with any missing value (include predictors and outcomes) |  | 4.5 Was the selection of predictors based on univariable analysis avoided? |
|  | 6.2 Number of participants with missing data for each predictor |  | 4.6 Were complexities in the data (e.g., censoring, competing risks, sampling of controls) accounted for appropriately? |
|  | 6.3 Handling of missing data (e.g., complete-case analysis, imputation, or other methods) |  | 4.7 Were relevant model performance measures evaluated appropriately? |
| 7. Model Development | 7.1 Modelling method (e.g., logistic, survival, neural network, or machine learning techniques) |  | 4.8 Were model overfitting and optimism in model performance accounted for? |
|  | 7.2 Modelling assumptions satisfied |  | 4.9 Do predictors and their assigned weights in the final model correspond to the results from multivariable analysis? |
|  | 7.3 Method for selection of predictors for inclusion in multivariable modelling (e.g., all candidate predictors, pre-selection based on unadjusted association with the outcome) |  | |
|  | 7.4 Method for selection of predictors during multivariable modelling (e.g., full model approach, backward or forward selection) and criteria used (e.g., p-value, Akaike Information Criterion) |  |  |
|  | 7.5 Shrinkage of predictor weights or regression coefficients (e.g., no shrinkage, uniform shrinkage, penalized estimation) |  |  |
| 8. Model Performance | 8.1 Calibration (calibration plot, calibration slope, Hosmer-Lemeshow test) and Discrimination (C-statistic, D-statistic, log-rank) measures with confidence intervals |  |  |
|  | 8.2 Classification measures (e.g., sensitivity, specificity, predictive values, net reclassification improvement) and whether a priori cut points were used |  |  |
| 9. Model Evaluation | 9.1 Method used for testing model performance: development dataset only (random split of data, resampling methods, e.g., bootstrap or cross-validation, none) or separate external validation (e.g., temporal, geographical, different setting, different investigators) |  |  |
|  | 9.2 In case of poor validation, whether the model was adjusted or updated (e.g., intercept recalibrated, predictor effects adjusted, or new predictors added) |  |  |
| 10. Results | 10.1 Final and other multivariable models (e.g., basic, extended, simplified) presented, including predictor weights or regression coefficients, intercept, baseline survival, model performance measures (with standard errors or confidence intervals) |  |  |
|  | 10.2 Any alternative presentation of the final prediction models, e.g., sum score, nomogram, score chart, predictions for specific risk subgroups with performance |  |  |
|  | 10.3 Comparison of the distribution of predictors (including missing data) for development and validation datasets |  |  |
| 11. Interpretation and Discussion | 11.1 Interpretation of presented models (confirmatory, i.e., model useful for practice versus exploratory, i.e., more research needed) |  |  |
|  | 11.2 Comparison with other studies, discussion of generalizability, strengths, and limitations |  |  |

Studies were stratified into three categories: (1) Conceptual tools, which proposed prognostic frameworks without original patient data or model development (e.g., expert-derived risk classifications, theoretical staging systems, or narrative frameworks describing prognostic domains); (2) Category-based prognostic tools, where predefined prognostic categories were assigned and subsequently validated against outcomes (e.g., “good/fair/poor” periodontal prognosis schemes, staging based on mobility/furcation, or fixed criteria validated against tooth loss); and (3) Data-driven prognostic models, ranging from conventional statistical approaches without external validation to modern statistical and machine-learning methods incorporating external validation (e.g., multivariable regression models predicting tooth loss, neural networks trained on clinical datasets, or externally validated machine-learning risk prediction tools).

## 2.6. Risk of bias (ROB) assessment

Three reviewers independently answered 20 questions within the PROBAST domains with predefined responses (Yes, Probably Yes, No Information, Probably No, No). Then, reviewers assessed the overall ROB and concerns for applicability, assigning ratings of Low, High, or Unclear for each domain. Disagreements were resolved by a fourth reviewer.

For the overall ROB in a domain:

- If all questions were answered as Y or PY, the domain was judged as "low ROB."
- Any N or PN flagged potential bias, but this did not confirm bias.
- NI indicated insufficient information, though assessors could still infer the ROB based on context (e.g., if predictors were measured before outcomes).

PROBAST was applied to assess risk of bias across all included studies. While its primary objective is to evaluate bias in estimates of model predictive performance, it can also be used for the critical appraisal of primary prediction model studies. In this context, its application allowed a structured assessment of methodological domains, even when performance metrics were not reported. For conceptual tools (i.e., studies without primary data), several PROBAST signaling questions, particularly those related to outcome determination, predictor assessment, and statistical analysis, could not be meaningfully addressed and were therefore classified as “not applicable” (NA). This designation indicates that the item is not relevant to the study design, rather than reflecting poor methodological quality or increased risk of bias. Similarly, for category-based models where patient data were available but not used to formally develop or estimate a prediction model, PROBAST domains related to participants, predictors, and outcomes were assessed as applicable, while items requiring model estimation, performance measures, or validation were classified as NA or NI, depending on the information provided.

## 2.7. Statistical Analysis

Descriptive analyses were conducted to summarize the distribution of ROB across studies and study designs. ROB assessments were derived from PROBAST signaling questions and summarized at the study level. Results are presented as proportions of studies rated as low, unclear, high, or not applicable across domains and study categories (Conceptual, Category-based, and Data-driven). Studies were categorized according to publication period (before vs. after 2014) to explore temporal trends following the introduction of TRIPOD. Given the structured and descriptive nature of PROBAST assessments, the presence of non-applicable items, and the limited number of studies within subgroups, no formal statistical comparisons between groups were performed.

# 3. Results:

## 3.1. Study selection and characteristics

### Selection

The initial electronic search identified 1345 records from PubMed, 225 from Embase, and 187 from Cochrane. After removing 152 duplicates, 1605 titles and abstracts were screened for eligibility. From these, 35 full-text articles were retrieved and assessed. Seventeen articles were excluded due to the following reasons: the prognostic tool was a secondary analysis of a previously published tool (Farina et al., 2021; Gopalakrishnan et al., 2024; Hasan et al., 2024; Ravidà, Qazi, et al., 2020; Saleh et al., 2023; Santamaria et al., 2024; Serroni et al., 2025); the study was a comparison among prognostic tools (Saleh et al., 2021; Saleh et al., 2022; Schwendicke et al., 2018); or only regression analysis was performed but no clinical tool was introduced (Chambrone & Chambrone, 2006; Dopico, Nibali, & Donos, 2016; Krois et al., 2019; Lee et al., 2023; Matuliene et al., 2008; Muzzi et al., 2006; Saminsky, Halperin-Sternfeld, Machtei, & Horwitz, 2015). Eighteen studies met the eligibility criteria and were included in the final evaluation. Citation searching identified an additional eleven records, of which two were excluded because no clinical tool was introduced (Furquim et al., 2025; Rebeiz et al., 2025). Nine studies were therefore included.

In total, 27 studies of periodontal prognostic tools designed for clinical application were included in the present systematic review. Supplementary Figure 1 presents the PRISMA flow diagram illustrating the selection process.

## Figure S1. PRISMA flowchart

**Identification of studies via other methods**

**Identification of studies via databases and registers**

Records identified from:

Citation searching (n = 11)

Records removed *before screening*:

Duplicate records removed (n = 152)

Records identified from:

Pubmed (n = 1345)

Embase (n = 225)

Cochrane (n = 187)

**Identification**

Records screened

(n = 1605)

Records excluded**

(n = 1570)

Reports not retrieved

(n = 0)

Reports sought for retrieval

(n = 11)

Reports sought for retrieval

(n = 35)

Reports not retrieved

(n = 0)

**Screening**

Reports assessed for eligibility

(n = 11)

Reports excluded:

Did not introduce a new clinical tool (n = 2)

Reports assessed for eligibility

(n = 35)

Reports excluded:

Secondary analysis of a previously published tool (n = 7)

Comparison among prognostic tools (n = 3)

Regression analysis but no prognostic tool (n = 7)

Studies included in review

(n = 27)

**Included**

*Consider, if feasible to do so, reporting the number of records identified from each database or register searched (rather than the total number across all databases/registers).

**If automation tools were used, indicate how many records were excluded by a human and how many were excluded by automation tools.

Source: Page MJ, et al. BMJ 2021;372:n71. doi: 10.1136/bmj.n71.

This work is licensed under CC BY 4.0. To view a copy of this license, visit <https://creativecommons.org/licenses/by/4.0/>

## Supplementary Table 3. Comparison of how primary studies were classified across the current review (Karaban et al.), and previous ones (Chow, Tay, & Nascimento, 2024; Du, Bo, Kapellas, & Peres, 2018). The present review included only studies that developed prognostic tools. Studies focused exclusively on tooth-loss prediction, diagnostic tools, or validation/modification of previously published models were not selected. Green cells indicate studies included in a given review, while red cells indicate exclusion. Studies published after the search periods of Chow et al. and Du et al. are shown on the right for reference.

| **Year** | **Study** | **Karaban et al.** | **Chow et al. 2024 (Chow et al., 2024)** | **Du et al. 2018** |
| --- | --- | --- | --- | --- |
| 1978 | Hirschfeld & Wasserman | Category-Based model | Not included | Not included |
| 1979 | Grant et al. | Conceptual tools | Not included | Not included |
| 1984 | Becker et al. | Category-Based model | Not included | Not included |
| 1991 | McGuire | Category-Based model | Not included | Not included |
| 1994 | Beck et al. | Not included: No tool developed | Not included | Prediction model *(not prognostic tool)* |
| 1996 | McGuire and Nunn | Not included: Modification/Validation of available tool | Validation study *(McGuire 1991)* | Not included |
| 1996 | Ghiai and Bissada | Category-Based model | Not included | Not included |
| 2002 | Checchi et al. | Category-Based model | Not included | Not included |
| 2002 | Gilbert et al. | Not included: No tool developed | Prediction model *(not prognostic tool)* | Not included |
| 2002 | Page et al. | Category-Based model | Not included | Prognostic tool |
| 2003 | Lang & Tonetti | Conceptual tools | Not included | Prognostic tool |
| 2003 | Page et al. | Not included: Modification/Validation of available tool | Validation study *(Page et al. 2002)* | Not included |
| 2004 | Fardal et al. | Category-Based model | Not included | Not included |
| 2007 | Faggion Jr et al. | Data-Driven model | Model Development study | Not included |
| 2007 | Kwok & Caton | Conceptual tools | Not included | Not included |
| 2008 | Jansson and Norderyd | Not included: Modification/Validation of available tool | Validation study *(Lang & Tonetti 2003)* | Not included |
| 2009 | Avila et al. | Conceptual tools | Not included | Not included |
| 2009 | Fan et al. | Not included: No tool developed | Prediction model *(not prognostic tool)* | Not included |
| 2009 | Martin et al. | Not included: Modification/Validation of available tool | Validation study *(Page et al. 2002)* | Not included |
| 2009 | Trombelli et al. | Conceptual tools | Not included | Prognostic tool |
| 2009 | Samet and Jotkowitz | Conceptual tools | Not included | Not included |
| 2010 | Leininger et al. | Not included: Modification/Validation of available tool | Validation study *(Lang & Tonetti 2003)* | Not included |
| 2010 | Lindskog et al. | Data-Driven model | Model Development study | Prognostic tool |
| 2010 | Martin et al. | Not included: Modification/Validation of available tool | Validation study *(Page et al. 2002)* | Not included |
| 2010 | Matuliene et al. | Not included: Modification/Validation of available tool | Validation study *(Lang & Tonetti 2003)* | Not included |
| 2011 | Carollo-Bittel et al. | Not included: Modification/Validation of available tool | Validation study *(Lang & Tonetti 2003)* | Not included |
| 2012 | Costa et al. | Not included: Modification/Validation of available tool | Validation study *(Lang & Tonetti 2003)* | Not included |
| 2012 | Meyer-Bäumer et al. | Not included: Modification/Validation of available tool | Validation study *(Lang & Tonetti 2003)* | Not included |
| 2012 | Nunn et al. | Data-Driven model | Model Development study | Not included |
| 2013 | Lü et al. | Not included: Modification/Validation of available tool | Validation study *(Lang & Tonetti 2003)* | Not included |
| 2014 | Levine et al. | Not included: No tool developed | Prediction model *(not prognostic tool)* | Not included |
| 2014 | Miller et al. | Data-Driven model | Model Development study | Not included |
| 2017 | Leite et al. | Not included: No tool developed | Not included | Prediction model *(not prognostic tool)* |
| 2017 | McGowan et al. | Conceptual tools | Not included | Not included |
| 2017 | Nibali et al. | Category-Based model | Model Development and Validation study | Not included |
| 2017 | Trombelli et al. | Not included: Modification/Validation of available tool | Validation study *(Trombelli et al. 2009)* | Not included |
| 2018 | Gopalakrishnan et al. | Not included: Modification/Validation of available tool | Validation study *(Miller et al. 2014)* | Not included |
| 2018 | Gopalakrishnan et al. | Not included: Modification/Validation of available tool | Validation study *(Miller et al. 2014)* | Not included |
| 2018 | Martinez-Canut and Llobell | Category-Based model | Model Development study | Not included |
| 2018 | Martinez-Canut et al. | Not included: No tool developed | Prediction model *(not prognostic tool)* | Prediction model *(not prognostic tool)* |
| 2018 | Morelli et al. | Not included: No prognostic tool developed | Diagnostic tool | Not included |
| 2018 | Morelli et al. | Not included: Modification/Validation of available tool | Validation study *(Morelli et al. 2018)* | Prediction model *(not prognostic tool)* |
| 2018 | Schwendicke et al. | Not included: No tool developed | Prediction model *(not prognostic tool)* | Not included |
| 2019 | Hirata et al. | Not included: Modification/Validation of available tool | Validation study *(Lü et al. 2013)* | Articles published after the review |
| 2019 | Meisel et al. | Not included: No tool developed | Prediction model *(not prognostic tool)* |  |
| 2019 | Ramseier et al. | Data-Driven model | Not included |  |
| 2020 | Ravidà et al. | Data-Driven model | Model Development and Validation study |  |
| 2020 | Shi et al. | Data-Driven model | Model Development study |  |
| 2021 | Farina et al. | Not included: Modification/Validation of available tool | Validation study *(Trombelli et al. 2009)* |  |
| 2021 | Nguyen et al. | Not included: Modification/Validation of available tool | Validation study *(Kwok & Caton 2007)* |  |
| 2021 | Saleh et al. | Not included: Modification/Validation of available tools | Validation study *(Grant et al. 1979; Becker et al. 1984; McGuire 1991; Checchi et al. 2002; Fardal et al. 2004; Faggion et al. 2007; Kwok & Caton 2007; Avila et al. 2009; Nunn et al. 2012; Miller et al. 2014)* |  |
| 2021 | Santos et al. | Data-Driven model | Model Development study |  |
| 2021 | Schwendicke et al. | Not included: No tool developed | Prediction model *(not prognostic tool)* |  |
| 2022 | Dharmarajan et al. | Not included: Modification/Validation of available tool | Validation study *(Miller et al. 2014)* |  |
| 2022 | Dukka et al. | Not included: Modification/Validation of available tool | Validation study *(Dietrich et al. 2019 - British New Classification)* |  |
| 2022 | El Sayed et al. | Not included: Modification/Validation of available tool | Validation study *(Papapanou et al. 2018 - New Classification)* |  |
| 2022 | Rahim-Wöstefeld et al. | Data-Driven model | Model Development study |  |
| 2022 | Saleh et al. | Not included: Modification/Validation of available tools | Validation study *(Page et al. 2002; Lang & Tonetti 2003; Trombelli et al. 2009; Tonetti et al. 2018)* |  |
| 2022 | Saydzai et al. | Not included: Modification/Validation of available tools | Validation study *(McGuire & Nunn 1996; Kwok & Caton 2007; Graetz et al. 2011; Nibali et al. 2017)* |  |
| 2022 | Takedachi et al. | Not included: Modification/Validation of available tool | Validation study *(Papapanou et al. 2018 - New Classification)* |  |
| 2022 | Troiano et al. | Data-Driven model | Model Development and Validation study |  |
| 2025 | Feher et al. | Data-Driven model | Article published after the review |  |

### Characteristics of included studies

Studies were stratified into:

(1) Conceptual tools (Avila et al., 2009; Grant, Stern, Everett, & Orban, 1979; Kwok & Caton, 2007; Lang & Tonetti, 2003; McGowan, McGowan, & Ivanovski, 2017; Samet & Jotkowitz, 2009; Trombelli, Farina, Ferrari, Pasetti, & Calura, 2009), which proposed prognostic frameworks but did not involve original patient data or model development;

(2) Category-based prognostic models (Becker, Becker, & Berg, 1984; Checchi, Montevecchi, Gatto, & Trombelli, 2002; Fardal, Johannessen, & Linden, 2004; Ghiai & Bissada, 1996; Hirschfeld & Wasserman, 1978; Martinez-Canut & Llobell, 2018; McGuire, 1991; Nibali et al., 2017; Page, Krall, Martin, Mancl, & Garcia, 2002), where predefined prognostic categories were assigned and subsequently validated against outcomes; and

(3) Data-driven prognostic models, with methodological refinement over time:

- Early studies(Faggion, Petersilka, Lange, Gerss, & Flemmig, 2007; Lindskog et al., 2010; Miller, McEntire, Marlow, & Gellin, 2014; Nunn et al., 2012) relied on conventional statistics to identify predictors, usually without external validation

- Intermediate studies(Ramseier et al., 2019; Ravidà, Troiano, et al., 2020; Santos et al., 2021; Shi et al., 2020) introduced nomograms and internal validation

- Most recent approaches(Feher et al., 2025; Rahim-Wöstefeld et al., 2022; Troiano et al., 2023) used advanced statistical or machine learning methods, often incorporating external validation

Six conceptual tools were proposed in narrative reviews (Avila et al., 2009; Grant et al., 1979; Kwok & Caton, 2007; Lang & Tonetti, 2003; McGowan et al., 2017; Samet & Jotkowitz, 2009). One study (Trombelli et al., 2009) had features of a retrospective cohort, as patients were included; however, its primary aim was not to develop or validate a prognostic model based on outcomes such as tooth loss or disease progression. Instead, the study assessed the performance of the PerioRisk tool by comparing it against another index. For this reason, the study was not classified as a fully data-driven prognostic investigation, but rather as a conceptual framework. All category-based models were retrospective cohort studies (Becker et al., 1984; Checchi et al., 2002; Fardal et al., 2004; Ghiai & Bissada, 1996; Hirschfeld & Wasserman, 1978; Martinez-Canut & Llobell, 2018; McGuire, 1991; Nibali et al., 2017; Page et al., 2002). Among data-driven prognostic models, most were retrospective cohort studies (Faggion et al., 2007; Feher et al., 2025; Miller et al., 2014; Nunn et al., 2012; Ramseier et al., 2019; Ravidà, Troiano, et al., 2020; Santos et al., 2021; Shi et al., 2020), while Lindskov et al.(Lindskog et al., 2010) and Rahim-Wöstefeld et al.(Rahim-Wöstefeld et al., 2022) were prospective and Troiano et al.(Troiano et al., 2023) included both prospective and retrospective external validation cohorts.

Tools operated at different units of prediction, including patient-level(Checchi et al., 2002; Fardal et al., 2004; Feher et al., 2025; Grant et al., 1979; Lang & Tonetti, 2003; Page et al., 2002; Ramseier et al., 2019; Ravidà, Troiano, et al., 2020; Santos et al., 2021; Trombelli et al., 2009), tooth-level(Avila et al., 2009; Becker et al., 1984; Faggion et al., 2007; Ghiai & Bissada, 1996; Hirschfeld & Wasserman, 1978; Kwok & Caton, 2007; Lindskog et al., 2010; Martinez-Canut & Llobell, 2018; McGowan et al., 2017; McGuire, 1991; Nibali et al., 2017; Nunn et al., 2012; Rahim-Wöstefeld et al., 2022; Samet & Jotkowitz, 2009), and molar-only(Miller et al., 2014; Shi et al., 2020; Troiano et al., 2023). One study reported a two-level prognostic framework. Lindskog et al. (2010) described a model in which patient-level risk assessment (Level I) was used to estimate the risk for chronic periodontitis, followed by tooth-level prognostication (Level II) to estimate the rate of disease progression based on marginal bone loss. Only the tooth-level model (Level II) was included in the present review.

Detailed characteristics of the studies, including enrolment period, study setting, study region, participants, modelling method, sample size, events, predictors, validation and performance measures are shown in Supplementary Tables 4 and 5.

## Supplementary Table 4. Comprehensive overview of participants characteristics, including number, male/female numbers, age, smokers.

| **Author, Year** | **Study design** | **Enrolment period** | **Study setting** | **Study region** | **Participant characteristics** | | | |
| --- | --- | --- | --- | --- | --- | --- | --- | --- |
|  |  |  |  |  | **Number (Male/Female)** | **Age of participants** | **Smokers** | **Periodontitis (type)** |
| Grant et al., 1979 | Narrative review | NA | NA | NA | NA | NA | NA | NA |
| Lang & Tonetti, 2003 | Narrative review | NA | NA | NA | NA | NA | NA | NA |
| Kwok & Caton, 2007 | Narrative review | NA | NA | NA | NA | NA | NA | NA |
| Avila et al., 2009 | Narrative review | NA | NA | NA | NA | NA | NA | NA |
| Samet & Jotkowitz, 2009 | Narrative review | NA | NA | NA | NA | NA | NA | NA |
| Trombelli et al., 2009 | Retrospective cohort | Not explicitly stated; retrospective data collection | Specialist periodontal clinic | Ferrara, Italy | 34/73 | 45.5 ± 9.9 | 45 never smoked, 18 former smokers, 44 current smokers | Not explicitly classified; presumed chronic periodontitis based on clinical parameters |
| McGowan et al., 2017 | Narrative review | NA | NA | NA | NA | NA | NA | NA |
| Hirschfeld & Wasserman, 1978 | Retrospective cohort | Recharting occurred between 1966 and 1971 | Private periodontal practice | Not explicitly stated, but likely New York (Columbia University affiliation) | 391(65.2%) / 209(34.8%) | 42 | NR | Categorized as early, intermediate, or advanced based on pocket depth |
| Becker et al.,1984 | Retrospective cohort | 5-13 years | Private periodontal practice. | Likely California, USA | 17 (39%) / 27 (61%) | 25-69 | NR | Moderate to severe periodontitis (generalized) |
| McGuire,1991 | Retrospective cohort | 2 months period of data selection, at least 5 years of follow-up No specific date range provided | No information | Texas, USA | 35 (35%) / 65 (65%) | 46.2 | 0 | NR |
| Ghiai & Bissada, 1996 | Retrospective cohort | 5-13 years | Case Western Reserve University | Cleveland, USA | 10 (41.7%) / 14 (58.3%)  580 teeth | NR | NR | Mild, moderate or severe periodontitis |
| Checchi et al., 2002 | Retrospective cohort | at least 4 years of follow-up, No specific date range provided | Private practice | Bologna, Italy | 37 (40%) / 75 (60%) | 45 | 0 | 9 (moderate) / 83 (advanced) |
| Page et al., 2002 | Prospective cohort | Not explicitly stated | University of Washington | Seattle, USA | 523 (men) | 49.6 | 19% smokers | NR |
| Fardal et al., 2004 | Retrospective cohort | 1997-1998 | dental practitioners, community dentists and physicians | 25 different dentists | 32 (32%) / 68 (68%) | 46 | 26 (26%) | Generalized mild, moderate or severe |
| Nibali et al., 2017 | Retrospective cohort | Treated between 2003–2009; last follow-up in June 2015 | Private periodontal practices | London and Bishop’s Stortford, UK | 40 (40%) / 60 (60%) | 53.04 ± 9.31 years | 22 current smokers, 20 former smokers, 58 never smokers | Moderate to severe chronic periodontitis |
| Martinez-Canut & Llobell, 2018 | Retrospective cohort | Follow-up 20–28 years (mean 24.7 ± 2.4); baseline period not explicitly stated | Private periodontal practice | Valencia, Spain | 34 (34%) / 66 (66%) | Mean 42.3 ± 6.95 (range 36–70) | NR | Moderate and severe chronic periodontitis |
| Faggion et al., 2007 | Retrospective cohort | 1 January 1989 - 31 December 1990 | Department of Periodontology, University of Münster and private practitoners | Münster, German | 95 (47.8%) / 103(52.2%) | 47.58 ± 10.42 | 0 | NR |
| Lindskog et al., 2010 | Prospective cohort | Baseline: Dec 1998–Mar 1999;  Follow-up: Oct–Dec 2002 | General dental and periodontal specialist clinics | Stockholm, Sweden | Baseline: 213 (55% female, 45% male); Follow-up: 183 (53% female, 47% male) | 47.9 | Categorized: <10, 10–20, >20 cigarettes/day; former smokers also recorded | Chronic periodontitis; stratified from no disease to severe |
| Nunn et al., 2012 | Retrospective study | No information  (previous studies) | Private practice | No information (previous studies) | NR | NR | NR | NR |
| Miller et al., 2014 | Retrospective cohort | 1969-1994 | Private dental practice | USA  (South Carolina) | 160 (102 male / 58 female) | 42 ± 9.47 | 33.3% | Moderate to severe |
| Ramseier et al., 2019 | Retrospective cohort | 1985-2011 | Dental hygiene school clinic | Bern, Switzerland | 394 (44.6%) / 489(55.4%) | 43.9 ± 13.0 | 30.3% smokers, 25.1% former smokers, 44.6% non-smokers | Categorized as mild, moderate, or advanced based on PPD profile |
| Ravidà et al., 2020 | Retrospective cohort | January 1966 - January 2007 | University of Michigan School of Dentistry | Michigan, USA | I - 124(48.82) and 130(51.18) II - 35(57.38) and 26 (42.62) | I - 46.72 ± 11.48; II - 51.41 ± 12.98 | 0 | Stage 2 and 3 at baseline in both cohorts (202/254, 79.53% in the development cohort and 48/61, 78.69% in the temporal validation cohort); similarly, the major number of patients were grade B (167/254, 65.75% in the development cohort and 41/61, 67.21% in the temporal validation cohort) |
| Shi et al., 2020 | Retrospective cohort | 1998-2009 | Department of Periodontology, Peking University School and Hospital of Stomatology | Peking | 80 (57.55%) / 59 (42.45%) | 41.8 ± 9.9 | 37 (27%) | 139 (100%) |
| Santos et al., 2021 | Retrospective cohort | May 2015 to 31 May 2018 | Periodontology department Of Egas Moniz Dental Clinic | Almada (Setúbal Penisula) | I (248/207); II (56/43) | I - 54.8 / II - 45.8 | I - 241(53%) / II - 44(44.4%) | stage I, II, III, IV |
| Rahim-Wöstefeld et al., 2022 | Prospective cohort | 1992 - 1997 | Section of Periodontology, Department of Conservative Dentistry | Heidelberg, Germany | 41 (38.2%) / 68 (61.8%) | 46.7 | former smokers  27/active 33 | GAP |
| Troiano et al., 2023 | Retrospective cohort | 1966 - 2020 | Universities | Two in Europe, one in the United States, one in China | 753 (51%) / 722 (49%) | 46.24 ± 11.65 | 669 (45%) | 222 (100%) |
| Feher et al., 2025 | Retrospective cohort | Data collected retrospectively from trials conducted before 2025 | South America | USA | 175(42%) / 239(58%) / 51(65%) / 35(%) | 45/48 | 0 | 0 |

GAP: Generalized aggressive periodontitis; LAP: Localized aggressive periodontitis; NA: not applicable; NR: not reported; PPD: probing pocket depth.

Cal: Calibration; Devel: Development; Disc: Discrimination; Ext: External validation; Int: Internal validation; Ov: Overall assessment; NR: No information; Valid: Validation.

## Supplementary Table 5. Comprehensive overview of model characteristics, including modeling method, sample size, number of predictors, selection of final predictors, missing data handling, type of validation, performance measures.

| **Author, Year** | **Tool** | **Sample size (patients/teeth)** | **Events**  **n (%)** | **Outcome** | **Modelling method** | **N Predictors**  **Cand.** | **N Predictors**  **Final** | **EPP; EPV** | **Selection of candidate predictors** | **Selection of final predictors** | **Number (%) and handling of missing data** | **Type of validation** | **Performance measures** |
| --- | --- | --- | --- | --- | --- | --- | --- | --- | --- | --- | --- | --- | --- |
| Grant et al., 1979 | Patient-level | NA | NA | Tooth loss | NA | 17 (Tooth therapeutic prognosis [% bone loss, pocket depth, distribution of bone loss, presence and depth of furcation involvements, mobility, crown-root ratio, pulpal involvement, tooth position and occlusal relationship, strategic value] and Overall prognosis [age, medical status, distribution and severity of tooth prognosis, degree of involvement/duration/history of the disease, patient cooperation, economic considerations, knowledge and ability of the dentist, etiologic factors) | NA | NA | Based on prior knowledge | NA | NA | NA | NA |
| Lang & Tonetti, 2003 | Patient-level | NA | NA | Disease progression | NA | 6 (BOP, PD ≥ 5 mm, tooth loss, BL/Age, Systemic/Genetic factors, Environmental factors) | NA | NA | Based on prior knowledge | NA | NA | NA | NA |
| Kwok & Caton, 2007 | Tooth-level | NA | NA | Stability/Disease progression | NA | 8 (patient compliance, cigarette smoking, diabetes mellitus, deep probing depth and attachment loss, other anatomic plaque-retentive factors, trauma from occlusion and parafunctional habits, mobility) | NA | NA | Based on prior knowledge | NA | NA | NA | NA |
| Avila et al., 2009 | Tooth-level | NA | NA | Tooth loss | NA | 27 (Initial assessment [patient expectations, treatment expectations, esthetics, finances, patients’ compliance], Periodontal disease severity [PD, mobility, recurrent periodontal abscess, bone loss, bone defect morphology], Furcation involvement [furcation, interproximal bone level to furcation entrance, root anomalies, root resection financial concerns], Etiologic factors [calculus, surgery compromises bone dimension, periodontal retreatment, root proximity, root canal therapy], Restorative factors [faulty restorations and fractures, extensive caries, crown:root, post/core and crown required], Other determinants [smoking, systemic conditions, use of bisphosphonates, clinician’s skill] | NA | NA | Based on prior knowledge | NA | NA | NA | NA |
| Samet & Jotkowitz, 2009 | Tooth-level | NA | NA | Tooth loss | NA | 4 (Periodontal health and alveolar support; Remaining tooth structure; Endodontic condition; Occlusal plane and tooth position) | NA | NA | Based on expert consensus and prior knowledge (Delphi method) | NA | NA | NA | NA |
| Trombelli et al., 2009 | Patient-level | 107 patients | NA | Tooth loss | Other | 5 (smoking status, diabetic status, PD ≥ 5mm, BOP, BL/Age) | NA | NA | Based on prior knowledge | Stepwise backwords | n (%): Unkown Method: No information | Int: None (Apparent performance) Ext : None | Cal: Not evaluated Disc : Not evaluated Ov: R-squared |
| McGowan et al., 2017 | Tooth-level | NA | NA | Tooth loss | NA | 9 (BL/Age, pocket depth, furcation involvement, infrabony defect, anatomic factors, mobility, smoking, diabetes, BOP) | NA | NA | Based on prior knowledge | NA | NA | NA | NA |
| Hirschfeld & Wasserman, 1978 | Tooth-level | 15666 teeth | 1100 (7.1%) | Tooth loss | NA | Favorable; Questionable: one or more of the following: furcation involvement (especially class II or III), deep, non-eradicable periodontal pockets, extensive alveolar bone loss (advanced radiographic loss of support), marked mobility (beyond physiologic range); Hopeless (teeth extracted before/during initial therapy) | NA | NA | NA | NA | n (%): Unkown Method: Complete-case analysis | NA | NA (outcomes reported descriptively: WM, D, ED groups; % questionable teeth lost; symmetry of tooth loss by type) |
| Becker et al.,1984 | Tooth-level | 1117 teeth | 67 (6%) | Tooth loss | NA | Good (Favorable): Adequate periodontal support, easy to maintain  Fair: About 25% attachment loss and/or class I furcation  Questionable: About 50% attachment loss, class II furcation (narrow), pockets 6–8 mm, unfavorable anatomy (grooves, furcations), extensive decay  Hopeless: >75% attachment loss, deep pockets >8 mm, class III furcation, class III mobility, poor crown–root ratio, root proximity with horizontal bone loss, recurrent abscesses | NA | NA | NA | NA | n (%): Radiographs missing in 11/44 patients Method: Complete-case analysis | NA | NA (descriptive: annual adjusted tooth loss 0.22 tooth/patient; prognosis-stratified loss: 3% of favorable lost, 37% of questionable lost, 33% of hopeless lost) |
| McGuire,1991 | Tooth-level | 2484 teeth | 181 (7.3%) | Tooth loss | NA | 1. Good Prognosis: Attachment / Bone: Adequate bone support, minimal to no attachment loss; Crown–root ratio: Favorable; Pocket depth: Shallow, easily maintainable; Furcation involvement: None; Mobility: None or physiologic; Tooth morphology: No significant root concavities, grooves, or proximity issues; Systemic / patient factors: No uncontrolled systemic disease, patient demonstrates good compliance and plaque control, non-smoker; Restorative factors: No significant caries, sound endodontic and restorative status; Maintenance: High likelihood of long-term stability with supportive periodontal therapy (SPT).  2. Fair Prognosis: Attachment / Bone: ~25% attachment loss; Pocket depth: Shallow to moderate, maintainable with routine care; Furcation involvement: Class I furcation; location and depth accessible for maintenance; Mobility: Slight (Class I); Crown–root ratio: Acceptable; Systemic / patient factors: Cooperative patient, systemic conditions under control; Restorative factors: Restorations possible without significant compromise; Maintenance: Tooth can be maintained, but prognosis is slightly less favorable than “Good.”  3. Poor Prognosis: Attachment / Bone: ~50% attachment loss; Crown–root ratio: Unfavorable but still functional; Pocket depth: Deeper pockets, more difficult to maintain; Furcation involvement: Class II; location and morphology make maintenance challenging; Mobility: Moderate (Class I–II); Tooth morphology: Presence of grooves, root concavities, or proximity increases risk; Systemic / patient factors: May be smoker or partially compliant, systemic health manageable but not ideal; Restorative factors: Tooth may have restorability issues (moderate decay, endo/restorative needs); Maintenance: Prognosis is guarded; tooth can be maintained short- to medium-term but long-term outcome uncertain.  4. Questionable Prognosis: Attachment / Bone: >50% attachment loss; Crown–root ratio: Poor; Pocket depth: Deep, non-maintainable sites; Furcation involvement: Advanced Class II (difficult access) or Class III; Mobility: ≥ Class II; Tooth morphology: Significant root proximity, deep concavities, unfavorable anatomy; Systemic / patient factors: May include poorly controlled systemic disease (e.g., diabetes), smoking, questionable compliance; Restorative factors: Endodontic and restorative concerns (caries, fractures, abutment failure); History: Recurrent periodontal abscesses or history of disease progression despite therapy; Maintenance: Tooth may be maintained short-term, but long-term survival is unlikely without advanced intervention.  5. Hopeless Prognosis: Attachment / Bone: >75% attachment loss; essentially inadequate support; Crown–root ratio: Non-functional; Pocket depth: Very deep, non-maintainable; Furcation involvement: Severe Class III (through-and-through, inaccessible); Mobility: Severe (Class III); Tooth morphology: Root proximity and anatomy unfavorable; Systemic / patient factors: Not decisive (tooth already unsalvageable); Restorative factors: Extensive caries, endodontic failure, fractured roots, failed abutment; History: Frequent abscesses or recurrent breakdown; Maintenance: Extraction indicated; tooth cannot be maintained in a functional, disease-free state. | NA | NA | NA | NA | n (%): 0 (0.0) Method: Complete-case analysis | NA | NA |
| Ghiai & Bissada, 1996 | Tooth-level | 580 teeth | 31 (5.3%) | Tooth loss | NA | Good: Alveolar bone score of > 50; Tooth mobility: 0 to 1; Clinical probing depth: less than 6 mm; Furcation involvement: none  Fair: Alveolar bone score of > 44 to 50; Tooth mobility: 1.5 to 2; Clinical probing depth: 6 to 8 mm; Furcation involvement: Class I  Guarded: Alveolar bone score of > 38 to 44; Tooth mobility: 2.5; Clinical probing depth: 8 to 10 mm; Furcation involvement: Class Il  Poor: Alveolar bone score of < 38; Tooth mobility: ≥ 3; Clinical probing depth: > 10 mm; Furcation involvement: Class III | NA | NA | NA | NA | n (%): 0 (0.0) Method: Complete-case analysis | NA | NA |
| Checchi et al., 2002 | Patient-level | 2310 teeth | 50 (2.2%) | Tooth loss | NA | Radiographic criteria:  Good: <50% bone loss  Questionable: 50–75% bone loss, or angular defect, or furcation involvement  Hopeless: >75% bone loss, or ≥2 questionable characteristics | NA | NA | NA | NA | n (%): 0 (0.0) Method: Complete-case analysis | NA | NA (descriptive) |
| Page et al., 2002 | Patient-level | 523 patients | 74% of men had at least one periodontally affected tooth at baseline | Disease progression | NA | Factors included: age, smoking, diabetes, history of periodontal surgery, pocket depth, bleeding on probing, subgingival restorations, root calculus, radiographic bone height, furcation involvement, vertical bone lesions  Score 1–2 = Low risk - Few risk factors, mild pockets, minimal bone loss, nonsmoker, no systemic disease.  Score 3 = Moderate risk - Some risk factors present (e.g., moderate PD, occasional BOP, smoker or diabetic but controlled).  Score 4 = High risk - Multiple risk factors, deeper pockets, more sites with BOP, radiographic bone loss, furcations.  Score 5 = Very high risk - Severe PD with generalized deep pockets, heavy smoking, uncontrolled diabetes, multiple restorations with subgingival margins, advanced bone/furcation/vertical defects. | NA | NA | NA | NA | n (%): 0 (0.0) Method: Complete-case analysis | NA | NA |
| Fardal et al., 2004 | Tooth-level | 2436 teeth | 58 (2.4%) | Tooth loss | NA | Good prognosis: Residual pocket depths (<3 mm) and <1/3 loss of proximal bone compared with normal bone height.  Uncertain prognosis: Residual pocket depths 4–6 mm with proximal bone loss of 1/3–2/3 of normal bone height. Inflammation of the tissues, with bleeding on probing. Where furcation involvement was present this did not exceed grade II.  Poor prognosis: Residual pocket depths ≥7 mm, proximal bone loss <2/3 of normal bone height. Inflammation of the tissues with bleeding on probing. Where furcation involvement was present it was at least class II. Horizontal mobility of up to 1 mm elicited at examination.  Hopeless prognosis: Pocket depths ≥9 mm. Inflammation of the tissues with bleeding on probing. Horizontal mobility of 41 mm with apical depressability and where furcation involvement was present it was of class III. If there was a question as to which prognosis a tooth should be given, the operator always assigned the most favourable prognosis. | NA | NA | NA | NA | n (%): 0 (0.0) Method: Complete-case analysis | NA | NA (descriptive: odds ratios) |
| Nibali et al., 2017 | Tooth-level | 2494 teeth | 45 (1.8%) | Tooth loss | NA | Age, gender, smoking, FMPS, FMBS, CAL, PRA prognosis, PRC prognosis, tooth prognosis (CAL, furcation, mobility, bone loss %, restorability, periapical pathology, prior endo).  Good prognosis: Minimal attachment/bone loss; no mobility or furcation; restorable, no endo issues  Fair prognosis: Moderate CAL/bone loss (≤30–40%), class I furcation, slight mobility, restorable, no endo problems  Poor prognosis: Advanced CAL/bone loss (≈50%), class II furcation, mobility ≥2, anatomical complications, restorative/endo concerns present  Hopeless prognosis: Severe CAL (>75%), non-restorable, or endodontic failure, class III furcation, severe mobility, recurrent abscess | NA | NA | NA | NA | n (%): 0 (0.0) Method: Complete-case analysis | NA | NA |
| Martinez-Canut & Llobell, 2018 | Tooth-level | NA | 219 | Tooth loss | NA | Addition of one score for each variable involved in the patient:  - Presence of subgingival calculus  - Gingival inflammation  - Vertical bone defects / Furcation involvement  - Gingival recession  - Abfractions | NA | NA | NA | NA | n (%): 0 (0.0) Method: Complete-case analysis | NA | NA |
| Faggion et al., 2007 | Tooth-level | 4559 teeth | 249 (5.5%) | Tooth loss | Logistic regression | 13 (diabetes, CHD, infectious disease, allergies, coagulation disorders, PPD, mobility, tooth type, API, SBI, bone level, pulp vitality, SPT frequency) | 5 (diabetes, bone level, mobility, root type, non-vital pulp) | 19.2 | Based on prior knowledge | Backward elimination | n (%): 0 (0.0) Method: Complete-case analysis    Smoking was not collected. | Int: None Ext : None | Cal: Not evaluated Disc : Log-rank test / Risk group curves / R-square Ov: R-squared |
| Lindskog et al., 2010 | Patient and tooth-level | 2928 at baseline; 2962 teeth at follow-up | 66 (2.3%) | Disease progression | Logistic regression | 20 (age, gender, smoking, diabetes, cardiovascular disease, number of teeth at baseline, plaque index, gingival index, probing depth, CAL, tooth type, mobility, furcation, crown/root ratio, root form, bone level, root proximity, endodontic involvement, caries, prosthetic status) | 20 (age, gender, smoking, diabetes, cardiovascular disease, number of teeth at baseline, plaque index, gingival index, probing depth, CAL, tooth type, mobility, furcation, crown/root ratio, root form, bone level, root proximity, endodontic involvement, caries, prosthetic status) | 3.3 | Based on prior knowledge | Pre-specified model (not selection) | n (%): NR Method: No imputation | Int: Other (repeated measures) Ext : None | Cal: Calibration plot  Disc : Logistic regression ORs; PPV, NPV, sensitivity, specificit Ov: R-squared |
| Nunn et al., 2012 | Tooth-level | 2509 teeth | 131 (5.2%) | Tooth loss | Machine learning techniques (Classification and Regression Trees [CART] for survival) | 22 (age, probing depth, furcation involvement, root form, crown/root ratio, mobility, smoking, type of bone loss, root proximity, oral hygiene, tooth malposition, fixed abutment, removable abutment, bite guard, parafunctional habit, parafunction + bite guard combo, % bone loss, compliance, family history, diabetes, endodontic involvement, caries) | 6 (furcation involvement, probing depth, crown/root ratio, age, mobility, mean % bone loss) | 6 | Based on prior knowledge | Other (automatic CART splits) | n (%): 0 (0.0) Method: Complete-case analysis | Int: Other (molars vs non-molars) Ext : None | Cal: Not evaluated Disc : Not evaluated Ov: Not evaluated |
| Miller et al., 2014 | Molar-only | 816 molars | 177 (21.7%) | Tooth loss | Cox regression | 6 (age, probing depth, mobility, furcation involvement, smoking, molar type) | 6 (age, probing depth, mobility, furcation involvement, smoking, molar type) | 29.5 | Based on prior knowledge | Pre-specified model (not selection) All retained | n (%): 0 (0.0) Method: Complete-case analysis | Int: None Ext : None | Cal: Calibration plot (Kaplan-Meier curves) Disc : C-Statistic / Risk group curves  Ov: Not evaluated |
| Ramseier et al., 2019 | Patient-level | 883 patients | 92 (20.7%) | Disease progression | Logistic regression / Linear mixed-effects regression | 6 (residual % PPD ≥4 mm, time between visits, BOP%, smoking, number of teeth, disease category at baseline [mild, moderate, severe]) | 3 (% PPD ≥4 mm at visit, time between visits, BOP%) | 15.3 | Based on prior knowledge | Other (significance testing in multivariable regression) | n (%): 0 (0.0) Method: Complete-case analysis | Int: Other (subgroup analysis, Kaplan–Meier) Ext : None | Cal: Calibration plot  Disc : OR for stability vs instability Ov: Not evaluated |
| Ravidà et al., 2020 | Patient-level | 315 patients (I - 254 develop and II - 61 validation) | I - 21 (8.3%) and II - 6 (9.6%) | Tooth loss | Logistic regression - Nomogram | 5 (stage, grade, extent, age, average SPT sessions/year) | 4 (stage, grade, extent, age) | 4.2 | Based on prior knowledge | Pre-specified model (not selection) | n (%): 0 (0.0) Method: Complete-case analysis | Int: Bootstrap Ext : Temporal validation | Cal: Calibration plot / Slope / CITL / HL test  Disc : C-Statistic / AUC graph  Ov: Measures of tendency and frequency |
| Shi et al., 2020 | Molar-only | 478 molars | 104 (21.8%) | Tooth loss | Cox regression - Nomogram | 12 (gender, age, probing depth, attachment loss, furcation involvement, BOP%, tooth mobility, mesial/distal bone level, root trunk length, vertical bone loss, bone loss area in furcation) | 9 (probing depth, attachment loss, furcation involvement, BOP, tooth mobility, mesial/distal bone loss, vertical bone loss, bone loss area in furcation) | 8.7 | Based on prior knowledge | Pre-specified model (not selection); Non-significant excluded | n (%): 0 (0.0) Method: Complete-case analysis | Int: Bootstrap Ext : None | Cal: Calibration plot / calibration curve Disc : C-Statistic / AUC graph / Time dependent Ov: Chi-square |
| Santos et al., 2021 | Patient-level | 544 patients (I: 455 development; II: 99 validation) | I: 103 (1.1%); II: 12 (0.6%) | Tooth loss | Logistic regression | 5 (smoking status, tooth type, probing depth, recession, clinical attachment loss) | 2 (tooth type, clinical attachment loss) | 20.6 | Based on prior knowledge | Stepwise logistic regression (p ≤ 0.25 entered, CAL + incisor retained) | n (%): 0 (0.0) Method: Complete-case analysis | Int: Non-random split data Ext : None | Cal: Calibration plot  Disc : C-Statistic / AUC graph  Ov: Mann–Whitney and Chi-squared tests |
| Rahim-Wöstefeld et al., 2022 | Tooth-level | 2556 teeth | 166 (6.5%) | Tooth loss | Generalized linear mixed model tree | 11 (age, gender, smoking, diabetes, plaque control record, jaw, tooth type, furcation involvement, interproximal bone loss, infrabony defect, abutment tooth) | 5 (bone loss, furcation involvement, abutment tooth, age, diabetes) | 15.1 | Based on prior knowledge | Bootstrap selection + Automated selection within GLMM-tree | n (%): 0 (0.0) Method: Complete-case analysis | Int: Cross-validation Ext : None | Cal: Not evaluated Disc : C-Statistic / AUC graph  Ov: Not evaluated |
| Troiano et al., 2023 | Molar-only | Development: 1475 molars; Validation: 1682 molars | Development: 77 (5.2%); Validation: 225 (13.4%) | Tooth loss | Machine learning techniques + Logistic regression | 9 (age, sex, smoking, furcation involvement, bone loss, probing depth, clinical attachment loss, mobility, abutment) | 9 (age, sex, smoking, furcation involvement, bone loss, probing depth, clinical attachment loss, mobility, abutment) | 8.6 | Based on prior knowledge | Pre-specified model (not selection) | n (%): London cohort had 7.1% missing Method: Complete-case analysis for most centers; London handled by simple imputation | Int: 10-fold cross-validation (with SMOTE oversampling in Michigan);  Ext = geographic (Beijing, Frankfurt, London cohorts) | Cal: Calibration plot / Slope / CITL  Disc : AUC graph / Risk group curves  Ov: Chi-square test |
| Feher et al., 2025 | Patient-level | Development: 414 patients; Validation: 78 patients | Development: 172 (42%); Validation: 32 (41%) | Clinical treatment success at 1 year (≤4 sites with PPD ≥5 mm) | Random forest algorithm | 18 (2 demographic: age, sex; 6 clinical: PPD, CAL, BoP, plaque, etc.; 7 microbiological complexes; 3 treatment-related: antibiotic type/dose/duration) | 18 (2 demographic: age, sex; 6 clinical: PPD, CAL, BoP, plaque, etc.; 7 microbiological complexes; 3 treatment-related: antibiotic type/dose/duration) | 9.6 | Based on prior knowledge | Pre-specified model (not selection); Relative importance analysis within RF | n (%): 0 (0.0) Method: Complete-case analysis | Int: Bootstrap and cross-validation Ext : Geographic | Cal: Calibration plot / Slope / CITL  Disc : AUC graph / F-1 score (Internal: 0.82, External: 0.71) Ov: Model achieved an out-of-bag score of 0.71 |

AUC (area under the ROC curve), BLA (bone loss area in furcation), BL (bone loss), BOP (bleeding on probing), CAL (clinical attachment level/loss), CART (classification and regression trees), CITL (calibration-in-the-large), C-statistic (concordance statistic), D-statistic (discrimination statistic), DBL (distal bone loss), Disc (discrimination), EPV (events per variable), EPP (events per parameter), F-1 score (harmonic mean of precision and recall), FMBS (full-mouth bleeding score), FMPS (full-mouth plaque score), GAP (generalized aggressive periodontitis), GLMM (generalized linear mixed model), GMCP (generalized moderate chronic periodontitis), GSCP (generalized stage II/III chronic periodontitis), HL test (Hosmer–Lemeshow test), LAP (localized aggressive periodontitis), LASSO (least absolute shrinkage and selection operator), LMCP (localized moderate chronic periodontitis), LSCP (localized stage II/III chronic periodontitis), MBL (mesial bone loss), NA (not applicable/available), NPV (negative predictive value), NR (not reported), OR (odds ratio), Ov (overall performance), PD (probing depth), PPD (probing pocket depth), PPV (positive predictive value), PRC (periodontal risk calculator), PRA (patient risk assessment), RF (random forest), ROC (receiver operating characteristic), RT (root trunk length), RuleFit (rule-based machine learning algorithm), SD (standard deviation), SMOTE (synthetic minority oversampling technique), SMOTEEN (SMOTE plus edited nearest neighbours), SPT (supportive periodontal therapy), RCT (root canal treatment), and VBL (vertical bone loss).

## Figure S2. Frequency distribution of responses (“Yes,” “No,” “Probably Yes”, “Probably No”, and “No Information”) for each PROBAST question across 20 studies in which PROBAST was applied.

## 3.2. Summary of included studies

An overview of the characteristics of the included tools is provided in Table 1. **Conceptual tools** (n = 7) consisted of narrative or review-based frameworks published between 1979 and 2017, proposing prognostic approaches without the use of original patient data or formal model development (Avila et al., 2009; Grant et al., 1979; Kwok & Caton, 2007; Lang & Tonetti, 2003; McGowan et al., 2017; Samet & Jotkowitz, 2009; Trombelli et al., 2009). Only two operated at the patient level (Lang & Tonetti, 2003; Trombelli et al., 2009), whereas the majority were tooth-level (5/7). The predicted outcomes were heterogeneous and often poorly defined: four tools referred broadly to disease progression (Lang & Tonetti, 2003; McGowan et al., 2017; Trombelli et al., 2009) or stability (Kwok & Caton, 2007) without specifying measurable parameters, while others addressed overall tooth loss (Samet & Jotkowitz, 2009), extraction decision-making (Avila et al., 2009), or overall therapeutic outcomes in a non-standardized manner (Grant et al., 1979). The prediction time was largely undefined (6/7), limiting interpretability; only one study incorporated explicit time frames (5-10 years)(McGowan et al., 2017), whereas the remaining tools did not specify when the predicted outcome would occur. Additionally, most tools (5/7) relied on predefined qualitative categories (e.g., low, moderate, high risk; or favorable to hopeless prognosis) without providing quantitative estimates. One tool also used categorical classification; however, the assignment of categories was largely dependent on clinician judgment due to the absence of explicit criteria (Kwok & Caton, 2007). The remaining tool was entirely qualitative, lacking structured categories or defined parameters (Grant et al., 1979). In terms of clinical application, patient-level tools were generally intended for use during re-evaluation or supportive periodontal therapy to guide maintenance intervals, whereas tooth-level tools were predominantly applied at 1^st^ examination. The format of presentation varied, including diagrams, tables, and narrative descriptions. Overall, these conceptual tools reflect the classical paradigm of periodontal prognostication, based on qualitative risk stratification rather than quantitative or algorithm-based prediction.

**Category-based prognostic tools** (n = 9) were derived from retrospective studies published between 1978 and 2018, where predefined prognostic categories were assigned and subsequently validated against outcomes (Becker et al., 1984; Checchi et al., 2002; Fardal et al., 2004; Ghiai & Bissada, 1996; Hirschfeld & Wasserman, 1978; Martinez-Canut & Llobell, 2018; McGuire, 1991; Nibali et al., 2017; Page et al., 2002). Only one tool operated at the patient level (Page et al., 2002), whereas the majority were tooth-level (8/9). The predicted outcomes were inconsistently defined. In several studies (5/9), the outcome of interest was not explicitly stated in the aims or during tool presentation; however, prognostic categories were subsequently correlated with periodontal (Checchi et al., 2002; Hirschfeld & Wasserman, 1978) or overall tooth loss (Becker et al., 1984; Ghiai & Bissada, 1996; McGuire, 1991). In contrast, four studies defined the prognostic tool predicted outcome a priori, including periodontal (Fardal et al., 2004) or overall tooth loss (Nibali et al., 2017), tooth survival (Martinez-Canut & Llobell, 2018), or disease progression (Page et al., 2002). Page et al. used disease progression as the predicted outcome, defined as radiographic bone loss > 2% across all included sites (patient-level tool). The prediction time was not explicitly defined in any study; although outcomes were assessed over follow-up periods, no tool specified the timeframe for prognostic estimation. Regarding methodology, most tools (7/9) relied on predefined categorical classifications (e.g., good to hopeless prognosis) based on clinical parameters. In contrast, one model applied an additive scoring system with equal weighting of variables to generate a risk scale (Martinez-Canut & Llobell, 2018), while another introduced a multivariable weighted algorithm producing a graded risk score (Page et al., 2002), reflecting a transition toward more quantitative approaches. In terms of clinical application, three tools were intended for use during re-evaluation to estimate risk during supportive periodontal therapy (Checchi et al., 2002; Fardal et al., 2004; McGuire, 1991), whereas the remainder were applied at the 1^st^ appointment. The majority of tools were presented descriptively within the text, typically prior to data analysis, with limited use of visual or digital formats. Overall, these models largely reflect the classical paradigm of prognostic categorization; however, unlike conceptual tools, they attempted to validate prognostic assignments against clinical outcomes within the same study, representing an initial step toward outcome-based prognostic modelling.

**Data-driven prognostic models** (n = 11) were derived from studies published between 2007 and 2025, with methodological refinement over time: Early studies(Faggion et al., 2007; Lindskog et al., 2010; Miller et al., 2014; Nunn et al., 2012) relied on conventional statistics to identify predictors, usually without external validation; Intermediate studies(Ramseier et al., 2019; Ravidà, Troiano, et al., 2020; Santos et al., 2021; Shi et al., 2020) introduced more structured multivariable approaches and improved model presentation (e.g., nomograms or equations), with limited or variable validation strategies; Most recent approaches(Feher et al., 2025; Rahim-Wöstefeld et al., 2022; Troiano et al., 2023) used advanced statistical or machine learning methods, often incorporating external validation. Across these models, prediction was performed at different levels, including patient-level (n = 4)(Feher et al., 2025; Ramseier et al., 2019; Ravidà, Troiano, et al., 2020; Santos et al., 2021), tooth-level (n = 4)(Faggion et al., 2007; Lindskog et al., 2010; Nunn et al., 2012; Rahim-Wöstefeld et al., 2022), and molar-specific models (n = 3)(Miller et al., 2014; Shi et al., 2020; Troiano et al., 2023). The predicted outcomes were more consistently defined compared to earlier approaches and predominantly focused on tooth loss or survival, either overall or cause-specific. Some models targeted periodontal tooth loss (Santos et al., 2021), while others evaluated overall tooth (Faggion et al., 2007; Nunn et al., 2012; Rahim-Wöstefeld et al., 2022) or molar (Miller et al., 2014; Shi et al., 2020; Troiano et al., 2023) loss or survival. Ravida et al. was a patient-level model predicting tooth loss (>1 tooth) due to periodontal causes. Additionally, studies addressing alternative outcomes, such as periodontal stability (Ramseier et al., 2019), disease progression (Lindskog et al., 2010), or treatment endpoints (Feher et al., 2025), provided explicit definitions of these parameters. The prediction time was more clearly specified compared to conceptual and category-based tools, with several studies defining fixed time points (e.g., risk for tooth loss after 1, 7, or 10 years, and up to 30 years in long-term models)(Feher et al., 2025; Miller et al., 2014; Rahim-Wöstefeld et al., 2022; Ravidà, Troiano, et al., 2020; Shi et al., 2020; Troiano et al., 2023). Other approaches used variable follow-up periods to derive predictions (Faggion et al., 2007; Lindskog et al., 2010; Nunn et al., 2012), while one model provided a dynamic framework, updating estimates during supportive periodontal therapy (Ramseier et al., 2019). In terms of output, most models generated quantitative probability estimates (0-100%) of the outcome of interest (Miller et al., 2014; Rahim-Wöstefeld et al., 2022; Santos et al., 2021; Shi et al., 2020; Troiano et al., 2023), while others transformed these probabilities into binary risk classifications (Feher et al., 2025; Ravidà, Troiano, et al., 2020) or used alternative formats such as scoring systems (Lindskog et al., 2010), categorical groupings (Faggion et al., 2007; Nunn et al., 2012), or threshold-based algorithms (Ramseier et al., 2019). Most models were intended for use at the first appointment. The presentation of models was heterogeneous, including nomograms, online tools, equations, graphical models, decision trees, and score-based indices, reflecting increasing efforts toward clinical applicability. Overall, these tools represent the transition and current state-of-the-art in periodontal prognostication, characterized by the use of multivariable models and algorithms to estimate individualized probabilities of outcomes or support clinical decision-making, rather than relying solely on qualitative risk categorization.

## 3.4. Applicability Assessment

Applicability was applied to category-based and data-driven models only.

### 3.4.1. Domain 1: Participants

Concerns regarding applicability in the Participants domain were low across all studies (Figure 2). Category-based models (Becker et al., 1984; Checchi et al., 2002; Fardal et al., 2004; Ghiai & Bissada, 1996; Hirschfeld & Wasserman, 1978; Martinez-Canut & Llobell, 2018; McGuire, 1991; Nibali et al., 2017; Page et al., 2002) and data-driven models (Faggion et al., 2007; Feher et al., 2025; Lindskog et al., 2010; Miller et al., 2014; Nunn et al., 2012; Rahim-Wöstefeld et al., 2022; Ramseier et al., 2019; Ravidà, Troiano, et al., 2020; Santos et al., 2021; Shi et al., 2020; Troiano et al., 2023) also presented study populations clearly aligned with the review question.

### 3.4.2. Domain 2: Predictors

In the Predictors domain, applicability concerns were predominantly rated as unclear across study designs (Figure 3). Category-based models (n=9) were judged as unclear concern as the predictor definitions used to form prognostic categories were heterogeneous across schemes and not not always specified in sufficient detail to determine whether they aligned with the predictors of interest, reflecting their methodology of first selecting the categories, and then testing for the outcome (Becker et al., 1984; Checchi et al., 2002; Fardal et al., 2004; Ghiai & Bissada, 1996; Hirschfeld & Wasserman, 1978; Martinez-Canut & Llobell, 2018; McGuire, 1991; Nibali et al., 2017; Page et al., 2002) the methodological practice of defining categories prior to outcome evaluation is addressed separately as a risk-of-bias concern in the Analysis domain and was not used to support the applicability rating. By contrast, data-driven models (n=11) demonstrated a more favorable profile: four were rated low concern (Lindskog et al. 2010, Santos et al. 2021, Rahim-Wöstefeld et al. 2022, Feher et al. 2025) while the remainder were judged unclear concern.

### 3.4.3. Domain 3: Outcome

In the Outcomes domain, applicability concerns varied across study designs were generally low (Figure 4). Category-based models (n=9) and data-driven models (n=11) were rated as low concern, as outcomes were directly aligned with the intended clinical application.

### 3.4.4. Overall Applicability

Overall, concerns regarding applicability were substantially lower than those observed for risk of bias (Figure 6). Conceptual tools (n=7) were “not applicable”, due to absent of empirical participants, measured predictors, and operationalized outcomes the absence of participants and poorly specified outcomes. In contrast, category-based models (n=9) were generally judged at unclear concern, with issues arising in the Predictors domain primarily reflecting heterogeneity in predictor specification across schemes. The most favorable profile was observed among data-driven models (n=11), where four studies were rated low concern across participants, predictors, and outcomes, and the remaining showed unclear concern. Taken together, participants, predictors, and outcomes evaluated in most studies were broadly applicable to the review question, indicating that applicability concerns were limited compared with the consistently high risk of bias observed across studies.

## 3.5. Reporting Standards

### 3.5.1. Methodological Improvements After TRIPOD Introduction: A Comparative Risk Analysis

ROB distribution across domains and time periods is presented in ROB distribution across PROBAST domains was compared between studies published ≤2014 (n=11: 7 category-based, 4 data-driven) and >2014 (n=9: 2 category-based, 7 data-driven)(Supplementary Figure 3). In the Participants domain, low risk was more frequent after 2014 (6/9 vs. 3/11), reflecting improvements in data source selection. In the Predictors domain, most studies in both periods were rated as unclear risk (9/11 ≤2014 vs. 6/9 >2014), primarily due to unreported blinding of predictor assessment. Only category-based studies published after 2014 (2/2) and a subset of data-driven studies (3/7) reported adequate predictor definition and assessment procedures. In the Outcome domain, unclear risk predominated in both periods (8/11 ≤2014 vs. 7/9 >2014). Low-risk ratings were more frequent after 2014 (2/9 vs. 1/11), reflecting improvements in outcome specification. In the Analysis domain, high risk predominated in both periods (10/11 ≤2014 vs. 8/9 >2014), driven by inadequate handling of missing data, underutilization of time-to-event methods, and limited internal validation. One recent study (Troiano et al., 2023) achieved low risk of bias through prospective design, adequate sample sizing, continuous variable handling, time-to-event analysis, and both internal and external validation.

To further contextualize these findings, studies reporting adherence to TRIPOD guidelines were examined separately. Among the four studies reporting TRIPOD adherence(Feher et al., 2025; Ravidà, Troiano, et al., 2020; Santos et al., 2021; Troiano et al., 2023), several methodological and reporting limitations remained across PROBAST domains. In the Predictors domain, blinding of predictor assessment was unclear in 50% of studies. In the Outcome domain, determination of outcomes without knowledge of predictor information was also unclear in 50% of studies, and one study showed high risk of bias due to inappropriate outcome definition. In the Analysis domain, all but one were rated as high risk of bias, mainly related to inadequate handling of model complexity, predictor selection, and lack of adjustment for overfitting or optimism. In contrast, reporting of participant selection and handling of missing data was generally adequate across studies.

## Figure S3. Proportions of studies with low (green), unclear (yellow), high (red), and not applicable (gray) ROB across the four domains (Participants, Predictors, Outcomes, Analysis) before and after 2014.

# References

Avila, G., Galindo-Moreno, P., Soehren, S., Misch, C. E., Morelli, T., & Wang, H. L. (2009). A novel decision-making process for tooth retention or extraction. *J Periodontol, 80*(3), 476-491. doi:10.1902/jop.2009.080454

Becker, W., Becker, B. E., & Berg, L. E. (1984). Periodontal treatment without maintenance. A retrospective study in 44 patients. *J Periodontol, 55*(9), 505-509. doi:10.1902/jop.1984.55.9.505

Chambrone, L. A., & Chambrone, L. (2006). Tooth loss in well-maintained patients with chronic periodontitis during long-term supportive therapy in Brazil. *J Clin Periodontol, 33*(10), 759-764. doi:10.1111/j.1600-051X.2006.00972.x

Checchi, L., Montevecchi, M., Gatto, M. R., & Trombelli, L. (2002). Retrospective study of tooth loss in 92 treated periodontal patients. *J Clin Periodontol, 29*(7), 651-656. doi:10.1034/j.1600-051x.2002.290710.x

Chow, D. Y., Tay, J. R. H., & Nascimento, G. G. (2024). Systematic Review of Prognosis Models in Predicting Tooth Loss in Periodontitis. *J Dent Res, 103*(6), 596-604. doi:10.1177/00220345241237448

Dopico, J., Nibali, L., & Donos, N. (2016). Disease progression in aggressive periodontitis patients. A Retrospective Study. *J Clin Periodontol, 43*(6), 531-537. doi:10.1111/jcpe.12533

Du, M., Bo, T., Kapellas, K., & Peres, M. A. (2018). Prediction models for the incidence and progression of periodontitis: A systematic review. *J Clin Periodontol, 45*(12), 1408-1420. doi:10.1111/jcpe.13037

Faggion, C. M., Jr., Petersilka, G., Lange, D. E., Gerss, J., & Flemmig, T. F. (2007). Prognostic model for tooth survival in patients treated for periodontitis. *J Clin Periodontol, 34*(3), 226-231. doi:10.1111/j.1600-051X.2006.01045.x

Fardal, Ø., Johannessen, A. C., & Linden, G. J. (2004). Tooth loss during maintenance following periodontal treatment in a periodontal practice in Norway. *J Clin Periodontol, 31*(7), 550-555. doi:10.1111/j.1600-051X.2004.00519.x

Farina, R., Simonelli, A., Baraldi, A., Pramstraller, M., Minenna, L., Toselli, L., . . . Trombelli, L. (2021). Tooth loss in complying and non-complying periodontitis patients with different periodontal risk levels during supportive periodontal care. *Clin Oral Investig, 25*(10), 5897-5906. doi:10.1007/s00784-021-03895-8

Feher, B., de Souza Oliveira, E. H., Mendes Duarte, P., Werdich, A. A., Giannobile, W. V., & Feres, M. (2025). Machine learning-assisted prediction of clinical responses to periodontal treatment. *J Periodontol*. doi:10.1002/jper.24-0737

Furquim, C. P., Caruth, L., Chandrasekaran, G., Cucchiara, A., Kallan, M. J., Martin, L., . . . Teles, F. (2025). Developing Predictive Models for Periodontitis Progression Using Artificial Intelligence: A Longitudinal Cohort Study. *J Clin Periodontol*. doi:10.1111/jcpe.14194

Ghiai, S., & Bissada, N. F. (1996). Prognosis and actual treatment outcome of periodontally involved teeth. *Periodontal Clin Investig, 18*(1), 7-11.

Gopalakrishnan, D., Miller, P. D., Levine, R. A., Sidharthan, S., Mahuli, A. V., Saleh, M. H. A., . . . Buranawat, B. (2024). Ten-year prognostic outcomes of molar survival using the Miller-Mcentire Periodontal Prognostic Index - A longitudinal prospective study. *J Indian Soc Periodontol, 28*(1), 75-78. doi:10.4103/jisp.jisp_542_23

Grant, D. A., Stern, I. B., Everett, F. G., & Orban, B. J. (1979). Periodontics in the tradition of Orban and Gottlieb. *(No Title)*.

Hasan, F., Magan-Fernandez, A., Akcalı, A., Sun, C., Donos, N., & Nibali, L. (2024). Tooth loss during supportive periodontal care: A prospective study. *J Clin Periodontol, 51*(5), 583-595. doi:10.1111/jcpe.13943

Hirschfeld, L., & Wasserman, B. (1978). A long-term survey of tooth loss in 600 treated periodontal patients. *J Periodontol, 49*(5), 225-237. doi:10.1902/jop.1978.49.5.225

Krois, J., Graetz, C., Holtfreter, B., Brinkmann, P., Kocher, T., & Schwendicke, F. (2019). Evaluating Modeling and Validation Strategies for Tooth Loss. *J Dent Res, 98*(10), 1088-1095. doi:10.1177/0022034519864889

Kwok, V., & Caton, J. G. (2007). Commentary: prognosis revisited: a system for assigning periodontal prognosis. *J Periodontol, 78*(11), 2063-2071. doi:10.1902/jop.2007.070210

Lang, N. P., & Tonetti, M. S. (2003). Periodontal risk assessment (PRA) for patients in supportive periodontal therapy (SPT). *Oral Health Prev Dent, 1*(1), 7-16.

Lee, C. T., Zhang, K., Li, W., Tang, K., Ling, Y., Walji, M. F., & Jiang, X. (2023). Identifying predictors of tooth loss using a rule-based machine learning approach: A retrospective study at university-setting clinics. *J Periodontol, 94*(10), 1231-1242. doi:10.1002/jper.23-0030

Lindskog, S., Blomlöf, J., Persson, I., Niklason, A., Hedin, A., Ericsson, L., . . . Blomlöf, L. (2010). Validation of an algorithm for chronic periodontitis risk assessment and prognostication: risk predictors, explanatory values, measures of quality, and clinical use. *J Periodontol, 81*(4), 584-593. doi:10.1902/jop.2010.090529

Martinez-Canut, P., & Llobell, A. (2018). A comprehensive approach to assigning periodontal prognosis. *J Clin Periodontol, 45*(4), 431-439. doi:10.1111/jcpe.12857

Matuliene, G., Pjetursson, B. E., Salvi, G. E., Schmidlin, K., Brägger, U., Zwahlen, M., & Lang, N. P. (2008). Influence of residual pockets on progression of periodontitis and tooth loss: results after 11 years of maintenance. *J Clin Periodontol, 35*(8), 685-695. doi:10.1111/j.1600-051X.2008.01245.x

McGowan, T., McGowan, K., & Ivanovski, S. (2017). A Novel Evidence-Based Periodontal Prognosis Model. *J Evid Based Dent Pract, 17*(4), 350-360. doi:10.1016/j.jebdp.2017.05.006

McGuire, M. K. (1991). Prognosis versus actual outcome: a long-term survey of 100 treated periodontal patients under maintenance care. *J Periodontol, 62*(1), 51-58. doi:10.1902/jop.1991.62.1.51

Miller, P. D., Jr., McEntire, M. L., Marlow, N. M., & Gellin, R. G. (2014). An evidenced-based scoring index to determine the periodontal prognosis on molars. *J Periodontol, 85*(2), 214-225. doi:10.1902/jop.2013.120675

Muzzi, L., Nieri, M., Cattabriga, M., Rotundo, R., Cairo, F., & Pini Prato, G. P. (2006). The potential prognostic value of some periodontal factors for tooth loss: a retrospective multilevel analysis on periodontal patients treated and maintained over 10 years. *J Periodontol, 77*(12), 2084-2089. doi:10.1902/jop.2006.050227

Nibali, L., Sun, C., Akcalı, A., Meng, X., Tu, Y. K., & Donos, N. (2017). A retrospective study on periodontal disease progression in private practice. *J Clin Periodontol, 44*(3), 290-297. doi:10.1111/jcpe.12653

Nunn, M. E., Fan, J., Su, X., Levine, R. A., Lee, H. J., & McGuire, M. K. (2012). Development of prognostic indicators using classification and regression trees for survival. *Periodontol 2000, 58*(1), 134-142. doi:10.1111/j.1600-0757.2011.00421.x

Page, R. C., Krall, E. A., Martin, J., Mancl, L., & Garcia, R. I. (2002). Validity and accuracy of a risk calculator in predicting periodontal disease. *J Am Dent Assoc, 133*(5), 569-576. doi:10.14219/jada.archive.2002.0232

Rahim-Wöstefeld, S., Kronsteiner, D., ElSayed, S., ElSayed, N., Eickholz, P., & Pretzl, B. (2022). Development of a prognostic tool: based on risk factors for tooth loss after active periodontal therapy. *Clin Oral Investig, 26*(1), 813-822. doi:10.1007/s00784-021-04060-x

Ramseier, C. A., Nydegger, M., Walter, C., Fischer, G., Sculean, A., Lang, N. P., & Salvi, G. E. (2019). Time between recall visits and residual probing depths predict long-term stability in patients enrolled in supportive periodontal therapy. *J Clin Periodontol, 46*(2), 218-230. doi:10.1111/jcpe.13041

Ravidà, A., Qazi, M., Troiano, G., Saleh, M. H. A., Greenwell, H., Kornman, K., & Wang, H. L. (2020). Using periodontal staging and grading system as a prognostic factor for future tooth loss: A long-term retrospective study. *J Periodontol, 91*(4), 454-461. doi:10.1002/jper.19-0390

Ravidà, A., Troiano, G., Qazi, M., Saleh, M. H. A., Lo Russo, L., Greenwell, H., . . . Wang, H. L. (2020). Development of a nomogram for the prediction of periodontal tooth loss using the staging and grading system: A long-term cohort study. *J Clin Periodontol, 47*(11), 1362-1370. doi:10.1111/jcpe.13362

Rebeiz, T., Lawand, G., Martin, W., Gonzaga, L., Revilla-León, M., Khalaf, S., & Megarbané, J. M. (2025). Development of an artificial intelligence model for assisting periodontal therapy decision-making: A retrospective longitudinal cohort study. *J Dent, 159*, 105780. doi:10.1016/j.jdent.2025.105780

Saleh, M. H. A., Dukka, H., Troiano, G., Ravidà, A., Galli, M., Qazi, M., . . . Wang, H. L. (2021). External validation and comparison of the predictive performance of 10 different tooth-level prognostic systems. *J Clin Periodontol, 48*(11), 1421-1429. doi:10.1111/jcpe.13542

Saleh, M. H. A., Dukka, H., Troiano, G., Ravidà, A., Qazi, M., Wang, H. L., & Greenwell, H. (2022). Long term comparison of the prognostic performance of PerioRisk, periodontal risk assessment, periodontal risk calculator, and staging and grading systems. *J Periodontol, 93*(1), 57-68. doi:10.1002/jper.20-0662

Saleh, M. H. A., Tattan, M., Troiano, G., Dukka, H., Ravidà, A., Levine, R., . . . Miller, P. D. (2023). Periodontal risk score: Initiation and model validation for 6,762 teeth. *J Periodontol, 94*(4), 459-466. doi:10.1002/jper.22-0273

Samet, N., & Jotkowitz, A. (2009). Classification and prognosis evaluation of individual teeth--a comprehensive approach. *Quintessence Int, 40*(5), 377-387.

Saminsky, M., Halperin-Sternfeld, M., Machtei, E. E., & Horwitz, J. (2015). Variables affecting tooth survival and changes in probing depth: a long-term follow-up of periodontitis patients. *J Clin Periodontol, 42*(6), 513-519. doi:10.1111/jcpe.12419

Santamaria, P., Troiano, G., Serroni, M., Araùjo, T. G., Ravidà, A., & Nibali, L. (2024). Exploring the accuracy of tooth loss prediction between a clinical periodontal prognostic system and a machine learning prognostic model. *J Clin Periodontol, 51*(10), 1333-1341. doi:10.1111/jcpe.14023

Santos, F., Beato, F., Machado, V., Proença, L., Mendes, J. J., & Botelho, J. (2021). Early Tooth Loss after Periodontal Diagnosis: Development and Validation of a Clinical Decision Model. *Int J Environ Res Public Health, 18*(3). doi:10.3390/ijerph18031363

Schwendicke, F., Schmietendorf, E., Plaumann, A., Sälzer, S., Dörfer, C. E., & Graetz, C. (2018). Validation of multivariable models for predicting tooth loss in periodontitis patients. *J Clin Periodontol, 45*(6), 701-710. doi:10.1111/jcpe.12900

Serroni, M., Ravidà, A., Santamaria, P., Dias, D. R., Cheng, G. L., Shaikh, S., . . . Troiano, G. (2025). Multi-Centre External Validation of a Nomogram for 10-Year Periodontal Tooth Loss Prediction. *J Clin Periodontol, 52*(7), 1044-1055. doi:10.1111/jcpe.14143

Shi, S., Meng, Y., Li, W., Jiao, J., Meng, H., & Feng, X. (2020). A nomogram prediction for mandibular molar survival in Chinese patients with periodontitis: A 10-year retrospective cohort study. *J Clin Periodontol, 47*(9), 1121-1131. doi:10.1111/jcpe.13343

Troiano, G., Nibali, L., Petsos, H., Eickholz, P., Saleh, M. H. A., Santamaria, P., . . . Ravidà, A. (2023). Development and international validation of logistic regression and machine-learning models for the prediction of 10-year molar loss. *J Clin Periodontol, 50*(3), 348-357. doi:10.1111/jcpe.13739

Trombelli, L., Farina, R., Ferrari, S., Pasetti, P., & Calura, G. (2009). Comparison between two methods for periodontal risk assessment. *Minerva Stomatol, 58*(6), 277-287.
